# Purine Chemistry in the Early RNA World at the Origins of Life: From RNA and Nucleobases Lesions to Current Key Metabolic Routes

**DOI:** 10.1002/cbic.202500035

**Published:** 2025-04-16

**Authors:** Jean‐Luc Décout, Marie‐Christine Maurel

**Affiliations:** ^1^ Département de Pharmacochimie Moléculaire UMR 5063 Université Grenoble Alpes CNRS Faculté de Pharmacie 38000 Grenoble France; ^2^ Institut de Systématique Evolution, Biodiversité (ISyEB) UMR 7205 CNRS Muséum National d’Histoire Naturelle Sorbonne Université 75005 Paris France

**Keywords:** Adenine, AICAR, histidine, hypoxanthine, metabolic pathways, origins and evolution of life, PRAMP, purine bases, RNA chemical modifications, RNA world, RNA/amino acid world

## Abstract

In early life, RNA probably played the central role and, in the corresponding RNA world, the main produced amino acids and small peptides had to react continuously with RNA, ribonucleos(t)ides and nucleobases, especially with purines. A RNA‐peptide world and key metabolic pathways have emerged from the corresponding chemical modifications such as the translation process performed by the ribosome. Some interesting reactions of the purine bicycle and of the corresponding ribonucleos(t)ides are performed under plausible prebiotic conditions and described RNA chemical lesions are reviewed with the prospect to highlight their connection with some major steps of the purine and histidine biosynthetic pathways that are, in an intriguingly way, related through two key metabolites, adenosine 5′‐triphosphate and the imidazole ribonucleotide 5‐aminoimidazole‐4‐carboxamide ribonucleotide. Ring‐opening reactions of purines stand out as efficient accesses to imidazole ribonucleotides and to formamidopyrimidine (Fapy) ribonucleotides suggesting that biosynthetic pathway’ first steps have emerged from RNA and ribonucleos(t)ide damages. Also, are summarized the works on the formation and catalytic properties, under plausible prebiotic conditions, of N6‐derivatives of the purine base adenine as potential surrogates of histidine in catalysis accordingly to their structural relationship.

## Graphical abstract

In the nascent processes of the beginnings and evolution of life, nucleobases and especially purines, ribonucleos(t)ides and primitive RNAs have been continuously modified. A RNA‐peptide world and key metabolic pathways probably have emerged from the corresponding chemical modifications resulting from adenine deamination, purine alkylation and, acylation from amino acids, purine ring‐opening reactions and rearrangements, RNA depurination and abasic site formation.

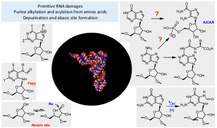

## 1 Introduction

How have emerged the first metabolic pathways? Is it possible to relate them to chemical reactions performed under plausible prebiotic conditions? In two recent review articles related to the chemistry at the origins of life, the authors pointed out the lack of experimental works relating the de novo ribonucleotide biosynthesis and the chemistry of abiotic nucleotide synthesis.^[^ ^1^, ^2^ ^]^ The review article focused on the chemistry of abiotic nucleotide synthesis also emphasized what remains to be achieved under prebiotic conditions and numerous questions without answers.^[^ ^1^ ^]^

**Figure 1:**
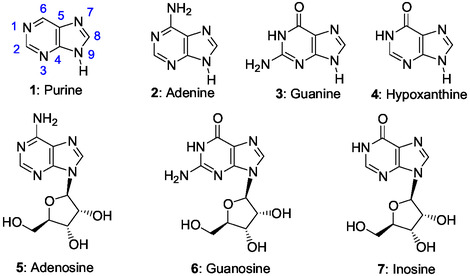
Structures of purine **1**, purine nucleic bases **2–4,** and their ribonucleosides **5–7** that are RNA elements.

Since the pioneer prebiotic synthesis of the purine (**1**) nucleic base adenine **2** performed by J. Oro in 1960 from ammonium cyanide in water,^[^ ^3^ ^]^ the abiotic synthesis of pyrimidine and purine (**1**) nucleobases, adenine **2** (**Figure** **1**), guanine **3**, and, hypoxanthine **4** that is formed in cells through RNA editing, was performed efficiently under plausible prebiotic conditions from different simple reagents such as hydrogen cyanide, urea, and formamide.^[^ ^2^, ^4^, ^5^, ^6^ ^]^ In 2012, a unified mechanism for abiotic synthesis of purine **1** and adenine **2** in formamide was proposed through formation of a critical glycine intermediate and imidazole derivatives, mechanism appearing to be reminiscent of the biosynthesis of purine nucleobases.^[^ ^6^ ^]^ However, the prebiotic synthesis of purine ribonucleos(t)ides **5**–**7** (Figure 1) by direct glycosylation of nucleic bases with ribose is considered as largely unsuccessful giving mixture of isomers often in low yields.^[^ ^1^ ^]^ Such a ribosylation is also considered as highly improbable since ribose is produced in the presumed prebiotic formose reaction (sugar formation from formaldehyde) as a minor product in the complex mixture of sugars formed.^[^ ^1^ ^]^ Already in 1914, it was clear that the formation of ribonucleosides by reaction of ribose with hypoxanthine, xanthine, adenine or guanine leads to the formation of the pyranosides instead of the furanosides.^[^ ^7^ ^]^ More recently, routes of synthesis of ribonucleosides and ribonucleotides were highlighted through the formation of reactive sugar intermediates obtained more efficiently than ribose under prebiotic conditions.^[^ ^1^, ^5^, ^8^, ^9^, ^10^, ^11^, ^12^, ^13^, ^14^, ^15^ ^]^ Moreover, such approaches have appeared to be much more difficult to achieve in good yields for the synthesis of purine ribonucleos(t)ides in comparison to the prebiotic synthesis of pyrimidine analogs. Another approach consisting to build the ribose ring from the nucleobases instead of combining both structures could be also possible, for example, in adapting, under prebiotic conditions, the synthetic chemistry recently developed for nucleoside synthesis.^[^ ^16^ ^]^ Such a prebiotic approach has been achieved with the synthesis of the four 2’‐deoxyribonucleosides found in DNA through formation and modification of N‐vinyl nucleobase derivatives (for more discussion see end of Part I and of Section).^[^ ^17^ ^]^

**Figure 2:**
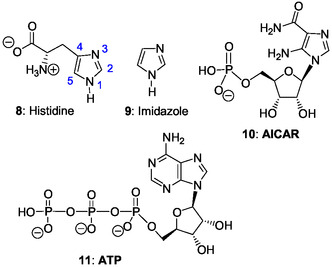
Structure of L‐histidine, imidazole, 1‐(5′‐phosphoribosyl)‐5‐amino‐4‐imidazolecarboxamide (AICAR), and ATP.

Among the numerous questions without answer, that of why the biosynthetic pathways of ribonucleotides and of the amino acid histidine **8** (**Figure** **2**) are closely related is key in view of the central roles of RNA in life evolution and histidine in enzymatic biocatalysis.^[^ ^18^, ^19^ ^]^

Purines metabolites are building blocks for RNA and DNA synthesis and provide the necessary energy and enzymatic cofactors for cell survival and proliferation. In the de novo biosynthesis of purine ribonucleotides (ribotides) and the biosynthetic pathway of histidine **8**, made of the imidazole ring **9**, we find the same intermediate 1‐(5′‐phosphoribosyl)‐5‐amino‐4‐imidazolecarboxamide **10** (AICAR) (Figure 2). AICAR is an imidazole ribonucleotide synthesized in both routes by nonhomologous enzymes, but used only for the purine biosynthesis.^[^ ^18^, ^19^ ^]^ Moreover, paradoxically, histidine is biosynthesized from adenosine 5’‐triphosphate (ATP) **11**. Therefore, such close connections between both pathways remain to be explained.

In a previous review article, these paradoxes were discussed as a case of functional molecular enzymatic convergence through the analysis of several different independent mechanisms of imidazole biosynthesis.^[^ ^19^ ^]^

In the first and second part of this review, we examined such a peculiarity from the rich chemistry of the purine bicycle in ribonucleos(t)ides and RNA illustrated by works performed under plausible prebiotic chemistry conditions. This chemistry suggests that hypoxanthine base **4** present in inosine and corresponding nucleos(t)ides are early key histidine precursors in a primitive nonenzymatic version of the de novo biosynthesis of purine ribotides. Deamination of nucleobases in DNA and RNA is a result of spontaneous hydrolysis, due to endogenous or environmental factors as well as deaminase enzymes.^[^ ^20^ ^]^ In RNA, formation of hypoxanthine from adenine **2** is an essential modification introduced by enzymes in a highly regulated manner to generate transcriptome diversity while presence of hypoxanthine in DNA corresponds to a damage. Recently, hypoxanthine in inosine has also appeared to be a key RNA base in the RNA self‐replication process that is an essential property for the emergence of life to copy and retain information.^[^ ^21^ ^]^ Surprisingly, an activated inosine ribonucleotide exhibits rapid and accurate nonenzymatic RNA copying as a surrogate of guanosine engaging non‐Watson–Crick base pairing.

In the third part of this review, we summarized our works on the catalytic properties of N6‐ adenine derivatives and, plausible prebiotic reactions of adenine and adenosine with pyruvaldehyde (methylglyoxal) giving N1,N6‐cyclic adducts. In N6‐substituted adenine derivatives, the 6‐aminopurine bicycle could have replaced the imidazole ring of histidine in an early catalysis since adenine is an amphoteric molecule that can act in aqueous solution as a relay for double proton transfer.

In this introduction, it is therefore necessary to specify the foundations and characteristics of the “RNA World” assumption.

*The “RNA World”*

The de novo biosynthesis of purine ribonucleotides has to be related to the “RNA World” hypothesis proposed by Gilbert.^[^ ^22^ ^]^ This RNA world, suggested initially by Woese, Crick, and Orgel, corresponds to an early biochemical period that would have existed before the contemporary DNA‐RNA‐Protein world.^[^ ^23^, ^24^, ^25^, ^26^ ^]^ In this early “mode” of life, RNAs probably assumed to be the central macromolecules, able to self‐replicate through base pairing, to retain information, and to catalyze the reactions necessary to a primitive metabolism alone and in cooperation with cofactors such as those used nowadays by protein enzymes.^[^ ^27^ ^]^ Unlike DNA, RNAs are usually present in cells as short or long monostrands. By demonstrating the remarkable molecular diversity of RNAs, molecular biology proved these predictions, through the discovery of RNA catalysts named ribozymes that catalyze RNA cleavage and splicing^[^ ^28^, ^29^ ^]^ or non‐natural reactions.^[^ ^30^, ^31^, ^32^ ^]^ RNA aptamers able to bind strongly to specific target due to their particular secondary and tertiary structures were also selected artificially.^[^ ^30^ ^]^ Natural RNA aptamers named riboswitches intervene in the regulation of various biological processes such as transcription and translation.^[^ ^33^, ^34^ ^]^ Therefore, RNA aptamers and ribozymes able to modulate metabolism and to catalyze diverse reactions such as redox transformations should have play a major role in the RNA world.^[^ ^35^, ^36^, ^37^, ^38^ ^]^ RNA present in modern cells performs structural and metabolic functions. A modern « RNA World » exists in each cell containing RNAs in various forms, short and long fragments, single and double‐stranded, endowed with multiple roles (informational, catalytic, as templates, guides, defense, etc.). Synthesized (transcribed) in the nucleus of eukaryote cells, after splicing, mature messenger RNAs (mRNAs), transfer RNAs (tRNAs), and ribosomal RNAs (rRNAs) are exported for translation as single strands to the cytoplasm of the cell after various maturation steps. Noncoding RNA transcripts such as introns resulting of the splicing process and other noncoding micro‐ and macro‐RNAs are also epigenetic regulators of gene expression. The mRNA and tRNA maturation process extends the diversity of RNA chemical structures through, for example, post‐transcriptional biochemical modifications that produce modified bases such as hypoxanthine and *N*6‐methyladenine.^[^ ^39^, ^40^ ^]^ *N*7‐methyl guanosine and 2’‐*O*‐methyl ribotides also constitute the mRNA cap allowing mRNA exportation into the cytoplasm of eukaryote cells. Bacteriophage genomes harbor the broadest chemical diversity of nucleobases across all life forms.^[^ ^41^ ^]^ Of these, 2,6‐diaminopurine (2‐aminoadenine), initially found in the cyanophage S‐2L DNA genome, pairs with thymine by forming three hydrogen bonds.^[^ ^41^, ^42^, ^43^ ^]^ The presence of modified canonic bases into contemporary genomes and RNAs as well as the diversity of pyrimidines and purines provided by abiotic chemistry suggests the existence of numerous noncanonical bases in primitive RNAs.^[^ ^39^, ^40^, ^41^, ^42^, ^43^, ^44^ ^]^

The early occurrence of a RNA world is supported by numerous metabolic facts. For instance, in the biosynthesis of DNA, dTMP is synthesized from dUMP that is methylated by thymidylate synthases with the cofactor methylene tetrahydrofolate as both a source of methylene and a reducing hydride^[^ ^45^ ^]^ or with a riboflavin cofactor^[^ ^46^ ^]^ as a methylene transporter and a reducing agent. 2’‐Deoxyribonucleotides (5’‐diphosphates and 5’‐triphosphates), including 2’‐deoxyuridine nucleotides and not the corresponding thymidine 2’‐deoxynucleotides, are produced through radical reduction of ribonucleotides by ribonucleotide reductases. Ribonucleotide reductase ancestors were probably close to the anaerobic *E. coli* ribonucleoside triphosphate reductase of class III that is a glycyl radical enzyme.^[^ ^47^ ^]^

*The “RNA/amino Acid World”*

Connections between the chemistry of purines and the chemistry of amino acids into RNA have offered the opportunity of molecular evolution leading to first metabolism routes from a primitive RNA‐amino acids world. Purine bases, purine nucleos(t)ides and RNA are nucleophiles that react with electrophiles. Electrophiles such as amino acid anhydrides^[^ ^48^, ^49^ ^]^ and lactones are thus potential candidates to generate chemical links between purine ribonucleotides/RNA and amino acids/peptides. Aspartic and related peptides anhydrides^[^ ^49^ ^]^ are good examples of potential electrophiles (see Section The Dimroth rearrangement in N1‐alkylated adenine and adenosine derivatives, and in N6‐acylated adenine derivatives) able to react with purines under plausible prebiotic conditions. Aminoacyl t‐RNAs and the corresponding translation process^[^ ^50^ ^]^ are vestiges of such a chemistry.^[^ ^51^, ^52^, ^53^, ^54^, ^55^, ^56^, ^57^, ^58^, ^59^, ^60^, ^61^, ^62^ ^]^

In ancestor RNAs, the close proximity of nucleic bases to emerging reagents such as amino acid and small peptide derivatives would have resulted in molecular damages^[^ ^40^, ^51^, ^52^, ^53^, ^54^, ^55^, ^56^, ^57^, ^58^, ^59^, ^60^, ^61^, ^62^ ^]^ like in modern RNA^[^ ^63^, ^64^, ^65^ ^]^ and DNA. Here, we use the word “damages” to point out all possible RNA chemical modifications that may have occurred in the nascent processes of the beginnings and evolution of life concomitantly with emergence of new reagents and catalysts in the corresponding environment. N‐alkylation of purines favors opening of the pyrimidine or imidazole ring and can result in deglycosylation of the produced nucleotides and in the formation of reactive abasic sites into RNA and DNA (see Part II). Further chemical modifications can be achieved into RNA at the same site from a primary purine damage since 1) the presence of the 2’‐hydroxyl group in purine ribonucleosides strongly slows down their deglycosylation in comparison to the corresponding 2’‐deoxyribonucleosides under acidic conditions;^[^ ^66^ ^]^ and 2) reactive abasic sites resulting from deglycosylation are more stable in RNA than in DNA under different conditions.^[^ ^67^ ^]^ Thereby, such processes could be at the origins of primeval metabolic‐like pathways taking place from and with RNAs. In following evolution steps, the release of modified RNA elements of metabolic interest may take place through depurination producing abasic sites and/or RNA cleavage induced by hydrolysis under basic conditions or catalyzed by metal ions, for instance. RNA deamination of N6‐modified cytosine and adenine derivatives, similar to the slow hydrolysis of adenine to give hypoxanthine,^[^ ^68^ ^]^ might also generate potential metabolites and peptides built on the purine bases. Therefore, the abiotic purine chemistry should enlighten the origins of key steps of the biosynthetic purine ribonucleotide and histidine pathways.

In cells, tRNAs and amino acids are covalently linked by a reactive ester function to form aminoacyl tRNAs from amino acid and ribose hydroxyl functions of a terminal adenosine. The ester function lability allows peptide bond formation and translation. Amino acids conjugated to purine and pyrimidine bases are also present in tRNAs as odd bases that may be seen as molecular fossils and useful for peptide synthesis (see Part II Section Odd bases in tRNAs and molecular fossils: adenosine N6‐carbamoylation and purine ribonucleotide N‐methylation).^[^ ^40^, ^51^, ^60^, ^69^, ^70^ ^]^ Many different chemical groups probably have linked nucleic bases to amino acids allowing transient cooperative chemical modifications (see Part II Section The Dimroth rearrangement in N1‐alkylated adenine and adenosine derivatives, and in N6‐acylated adenine derivatives) and catalysis under abiotic conditions. The chemical evolution of produced primary RNA‐amino acid adducts should have been central in the metabolism development.

Hereinafter, we examine some interesting reports on purine reactions and related RNA lesions formed under mild conditions with the perspective of their potential role in the emergence of the purine ribonucleotide metabolism and some other key metabolic pathways such as the biosynthetic route giving histidine that has a purine‐related structure.

## 2 Part I. Biosynthetic Pathways of Purine Ribonucleotides and Histidine, First Relationships

### 2.1 The Biosynthesis of Purine Ribonucleotides

**Scheme 1:**
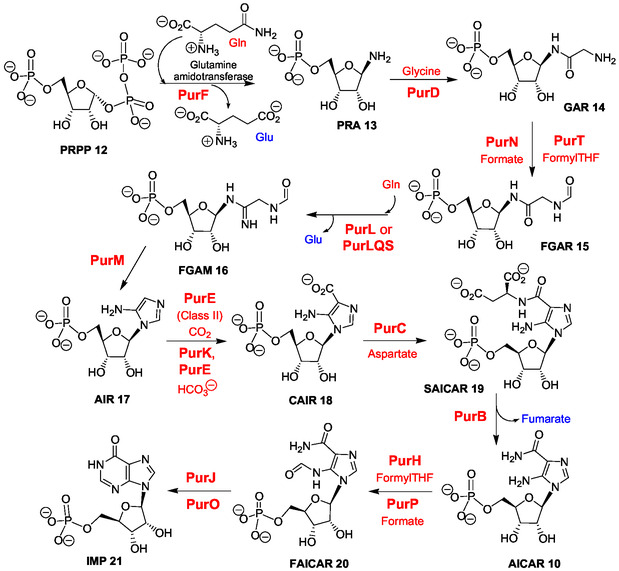
Biosynthetic pathway of IMP (Adapted with permission.^[^ ^72^ ^]^ Copyright, The KEGG database).

**Figure 3:**
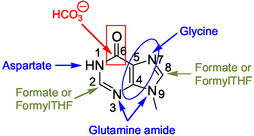
Biosynthetic origins of the purine bicycle atoms. Blue color corresponds to atoms of amino acids; FormylTHF: 10‐formyltetrahydrofolate enzymatic cofactor.

**Scheme 2:**
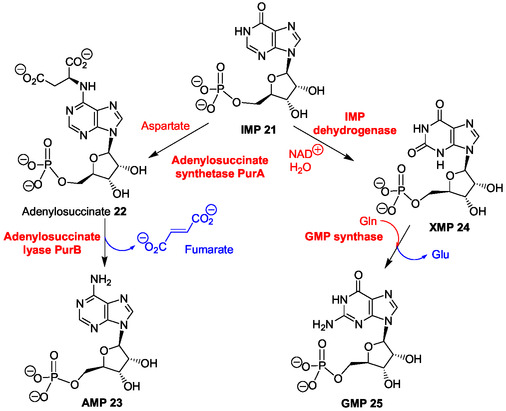
Biosynthetic pathway of AMP and GMP (Adapted with permission.^[^ ^73^ ^]^ Copyright, The KEGG database).

The de novo purine ribonucleotide biosynthetic pathway is based on the biosynthesis of inosine 5’‐phosphate (IMP **21**) and was reviewed accurately to highlight different topics including enzyme mechanisms, protein evolution, and drug design.^[^ ^71^, ^72^, ^73^ ^]^ The biosynthesis of IMP and the corresponding origins of hypoxanthine atoms are summarized in **Scheme** **1** and **Figure** **3**, respectively. IMP biosynthesis requires ten enzymatic steps and, then, adenosine 5’‐monophosphate (AMP **23**) and guanosine 5’‐monophosphate (GMP **25**) are produced separately from IMP in two steps (**Scheme** **2**). In the IMP biosynthesis, the enzymes PurF, PurD, PurL, PurM, PurC, and PurB are common to all IMP pathways, while PurN or PurT, PurK/PurE‐I or PurE‐II, PurH or PurP, and PurJ or PurO catalyze the same steps in different organisms.^[^ ^72^ ^]^ Ancestral proteins may have included a broad specificity enzyme instead of PurD, PurT, PurK, PurC, and PurP, and a separate enzyme instead of PurM and PurL.^[^ ^72^ ^]^ The molecular evolution of the pathway has been reviewed through structural studies, sequence alignments, biochemistry, and chemistry with emphasis on the binding of the ribose 5’‐phosphate moieties, common to all purine biosynthetic intermediates, and the transient protein–protein interactions in channeling of chemically unstable intermediates.^[^ ^71^ ^]^

The purine bicycle is built from unstable 5‐phosphoribosylamine (PRA **13**)^[^ ^71^, ^74^ ^]^ and via five imidazole ribonucleoside 5’‐monophosphate intermediates (ribotides) (Scheme 1). In the first step, 5‐phosphoribosyl‐1‐pyrophosphate (PRPP **12**) is converted to PRA through deamination of glutamine used as an ammonia donor. Recently, PRPP was obtained under plausible prebiotic conditions on silica gel from ribose and potassium dihydrogenophosphate and led in the presence of adenine **2** to AMP **23**.^[^ ^75^ ^]^ In the following three steps of IMP biosynthesis, a side chain is introduced on the PRA amine function by condensation with glycine and, then, modified to build the imidazole ring (formation of **14**–**16**). In the six next steps, imidazole ribotide intermediates are successively synthesized, 5‐amino‐imidazole ribotide (AIR **17**), 4‐carboxy‐5‐aminoimidazole ribonucleotide (CAIR **18**), 4‐(*N*‐succinylo)‐carboxamido‐5‐aminoimidazole ribotide (SACAIR **19**), 5‐aminoimidazole‐4‐carboxamido ribotide (AICAR **10**), and 5‐formamido‐1‐(5‐phosphoribosyl)imidazole‐4‐carboxamido ribotide (FAICAR **20**) (Scheme 1). A final cyclization step allows the formation of the hypoxanthine bicycle of IMP **21**.

Both main purine ribonucleotide 5’‐monophosphates incorporated in RNA from the corresponding 5’‐triphosphates are biosynthesized from IMP **21** that is converted to AMP **23** by succinylation with aspartate giving adenylosuccinate **22** (PurA enzyme) and, then, through fumarate elimination (PurB enzyme) (Scheme 2).^[^ ^73^, ^76^, ^77^, ^78^ ^]^ GMP **25** is obtained in two steps by oxidation of hypoxanthine base to give the xanthine bicycle of XMP **24** followed by amination at position 2 using glutamine as ammonia donor (Scheme 2). As mentioned in the introduction, a chemical purine hybrid between adenine and guanine, 2,6‐diaminopurine (2‐aminoadenine, nucleobase Z), replaces adenine in the DNA of the cyanobacterial virus *Synechococcus* phage S‐2L and Vibrio phage PhiVC8.^[^ ^41^, ^42^, ^43^ ^]^ S‐2L and PhiVC8 encode a third purine pathway catalyzed by PurZ, a distant paralog of succinoadenylate synthase (PurA, Scheme 2). PurZ condenses aspartate with deoxyguanylate into *N*6‐succino‐2‐amino‐2′‐deoxyadenylate, which undergoes defumarylation and phosphorylation to give dZTP (2‐amino‐2′‐deoxyadenosine 5′‐triphosphate), a substrate for the phage DNA polymerase.^[^ ^43^ ^]^ Crystallography and phylogenetics analyses indicate a close relationship between phage PurZ and archaeal PurA enzymes.^[^ ^43^ ^]^

### 2.2 The Histidine Biosynthetic Pathway

Histidine **8** (Figure 2) is a key ubiquitous amino acid in enzymes that serves a central role in biochemical catalysis and as a ligand for metallic ion complexation in heme and nonheme enzymes, carrier proteins, and, transcription factors. Histidine plays a prominent role in the acid–base catalysis developed by many enzymes and is by far the most common amino acid found in the active site of enzymes.^[^ ^79^ ^]^ The key role of histidine residue in catalysis can be illustrated by the acid–base catalysis of RNA hydrolysis achieved with two imidazole rings by ribonucleases, catalysis that could have played a central role in the recovering for recombination of building blocks in the RNA world.

**Scheme 3:**
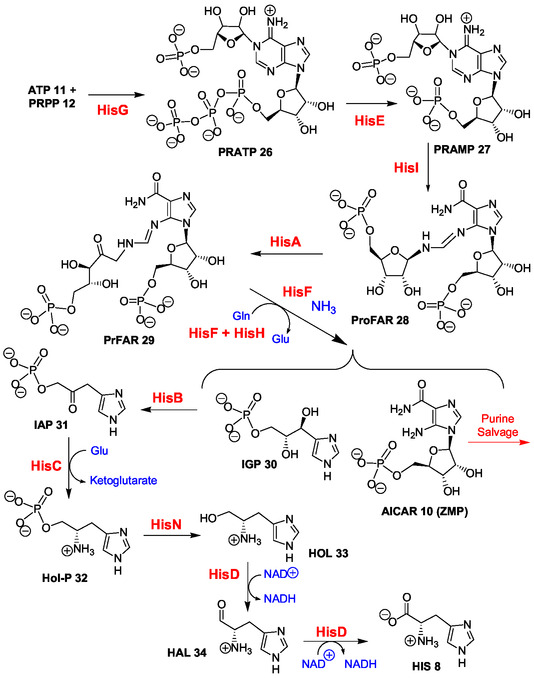
Biosynthetic pathway of histidine (Adapted with permission.^[^ ^18^, ^19^, ^80^, ^81^, ^82^ ^]^ Copyright, The KEGG database).

**Figure 4:**
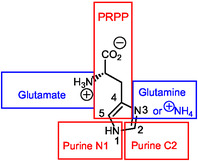
Biosynthetic origins of histidine atoms. The red color corresponds to atoms of adenine in ATP and the blue color corresponds to atoms of aminoacids.

The biosynthetic pathway of L‐histidine **8** and the corresponding atom origins are detailed in **Scheme** **3** and **Figure** **4**, respectively.^[^ ^18^, ^19^, ^80^, ^81^, ^82^ ^]^ L‐Histidine biosynthesis is an ancient metabolic pathway present in bacteria, archaea, lower eukaryotes, and plants. For decades, L‐histidine biosynthesis has been studied mainly in *E. coli* and *S. typhimurium*, revealing fundamental regulatory processes in bacteria. Furthermore, in the last 15 years, this pathway has been also investigated intensively in the industrial amino acid‐producing bacterium *C. glutamicum*, revealing similarities to *E. coli* and *S. typhimurium*, as well as differences.^[^ ^80^ ^]^ Since the late 1950s, the histidine biosynthesis pathway has also been studied intensively in different organisms like yeasts. Initially, Ames and Martin elucidated the complete histidine pathway by identifying all metabolic intermediates and the enzymes catalyzing the corresponding reactions in *S. typhimurium*.^[^ ^83^, ^84^ ^]^ At that time, last uncertainties remained regarding the reaction steps and intermediates at the interconnection to the pathway of de novo purine biosynthesis. These issues were finally elucidated revealing the final number of catalytic reactions and intermediates.^[^ ^85^ ^]^ Based on this knowledge, histidine biosynthesis is an unbranched pathway with ten enzymatic reactions starting with PRPP **12**.^[^ ^86^, ^87^ ^]^ It turned out early that the histidine pathways of *S. typhimurium* and *E. coli* were identical. Moreover, histidine biosynthesis seems to be conserved in all organisms including archaea,^[^ ^88^ ^]^ Gram‐positive bacteria,^[^ ^89^ ^]^ lower eukaryotes,^[^ ^90^ ^]^ and plants.^[^ ^87^ ^]^ The general histidine pathway and its regulation have been extensively reviewed, mainly focusing on *E. coli*, *S. typhimurium*, and plants.^[^ ^83^, ^84^, ^86^, ^87^, ^91^ ^]^ The histidine gene cluster of *E. coli* and *S. typhimurium* was one of the model gene clusters leading to the development and approval of the operon theory.^[^ ^86^ ^]^ In both organisms, all eight histidine biosynthesis genes are part of one operon and therefore transcribed and regulated as a single unit.^[^ ^92^, ^93^, ^94^ ^]^ This concentration of all histidine biosynthesis genes at one locus seems not to be the rule but rather an exception and restricted to the enterobacteria, since in other bacteria these genes are more scattered throughout the genome.^[^ ^86^ ^]^ The biosynthesis is achieved from the adenine base of ATP **11** and PRPP **12** (Scheme 3) that is also a precursor in the de novo synthesis of purine ribotides (Scheme 1). Carbon and nitrogen atoms of the histidine imidazole (im) ring have as origins PRPP (ribose: imC3 and imC4), ATP (adenine N1 and C2: imN1‐ and imC2, respectively) and ammonia or, alternatively, glutamate (imN3) (Figure 4).

### 2.3 Relationships between the Biosynthetic Pathways of Purine Ribotides and Histidine

In the recent review discussing the evolutionary convergence in the biosynthesis of the imidazole moieties of histidine and purines, the authors point out differences in the corresponding biosynthesis made by nonhomologous enzymes.^[^ ^19^ ^]^ They suggest that, in evolutionary terms, the purine biosynthesis predated that of histidine and highlights the diversity of imidazole‐synthesizing pathways emphasizing the biological importance of imidazole, whose biosynthesis evolved independently several times.

Recently, the order of amino acid recruitment into the genetic code appeared to be resolved by analysis of the last universal common ancestor's (LUCA's) protein domains and the emergence of histidine was discussed.^[^ ^95^ ^]^ The authors mentioned 1) that the recent classification of histidine as abiotically unavailable^[^ ^96^ ^]^ also contributed to its annotation as late; and 2), while histidine can be abiotically synthesized from erythrose reacting with formamidine followed by a Strecker synthesis reaction,^[^ ^97^ ^]^ the reactant concentrations might have been insufficient in a primitive earth environment.^[^ ^19^ ^]^ They concluded that histidine had been added to the genetic code earlier than expected from its molecular weight and underlined that, more importantly, because histidine resembles a purine, even if histidine were abiotically unavailable, it might have had cellular availability at the time of genetic code construction,^[^ ^98^ ^]^ in an organism that biotically synthesized ribosomes, and that might also have already utilized amino acids and peptides.^[^ ^79^ ^]^

In the de novo synthesis of purine ribonucleotides (Scheme 1), the purine bicycle is built from 5‐phosphoribosylamine (PRA **13**) and, then, via five imidazole ribotide intermediates. In the biosynthetic histidine pathway (Scheme 3), N1‐ribosylation in the adenine bicycle of ATP **11** produced the “byproduct” AICAR **10** usable in a purine salvage pathway (Figure 1).^[^ ^99^ ^]^ Interestingly, all atoms of AICAR originate from ATP except the oxygen atom of the 4‐amide function originating from a water molecule that induces adenine pyrimidine ring‐opening after ribosylation at N1 (see Section Key pyrimidine ring‐opening in the histidine biosynthetic pathway and abiotic pyrimidine ring‐opening in N1‐modified adenine ribos(t)ides). These origins of AICAR atoms bring out the major relationship between both pathways and strengthen the assumption of a purine ribotide biosynthesis predating histidine biosynthesis. The connections between both pathways through ATP and AICAR have led to the proposal that histidine is, in fact, the molecular vestige of an ancient catalytic nucleotide, part of the RNA world since it is the only imidazole‐bearing amino acid and known amino acid with a ribonucleotide‐starting biosynthesis.^[^ ^100^ ^]^

In the histidine pathway, the complex reaction of PrFAR **29** with ammonia (Scheme 3, step 5) is especially intriguing since it produces two imidazole derivatives, IGP **30** (imidazole glycerophosphate) and AICAR **10**.^[^ ^19^ ^]^ The corresponding reaction is performed from ammonia (ammonium ions) by the enzyme HisF or from glutamine by the enzymes HisF and HisF (HisFH).^[^ ^101^, ^102^, ^103^ ^]^ HisF seems to play a central role in cellular metabolism highlighting the interconnections of different metabolic pathways.^[^ ^102^ ^]^ Both produced imidazole derivatives **10** and **30** result from the nucleophilic attack of ammonia on the phosphoribulosylformimino (PrF) group of PrFAR inducing cleavage and intramolecular cyclization.^[^ ^19^ ^]^ Formally, PrFAR **29** is as an AICAR derivative carrying the PrF group attached to 5‐amino substituent of the imidazole ring. Two ring‐opening steps are involved in the formation of the PrF group: 1) pyrimidine ring‐opening of the adenine bicycle in PRAMP **27;** and 2) ribose ring‐opening and internal redox reaction in ProFAR **28**.

Transient protein–protein interactions in channeling of chemically unstable intermediates such as PRA **13** are important (5‐phosphoribosylamine showed a very short half‐life under physiological conditions) and Stubbe et al. have postulated that channeling could be important between PurF and PurD and between PurK and PurE.^[^ ^71^, ^74^ ^]^ This remark suggests that, in a primitive purine nucleotide synthetic pathway, probably, chemical cross‐reactions between metabolites or analogs should have been happened.

**Scheme 4:**
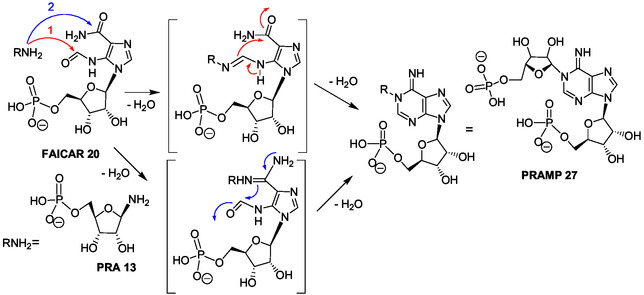
Potential reactions giving PRAMP **27** from PRA **13** and FAICAR **20** that are involved in the first and last step of the biosynthetic pathway of IMP **21**, respectively (Scheme 1). Route 1 corresponds to reaction of the N5‐formyl group (red arrow). Route 2 corresponds to reaction of the 4‐carboxamido substituent (blue arrow) and can also give the N6‐phosphoribosyl AMP isomer that may be converted to PRAMP **27** through a Dimroth rearrangement and inversely (see Part II Section The Dimroth rearrangement in N1‐alkylated adenine and adenosine derivatives, and in N6‐acylated adenine derivatives).

For example, formally, PRAMP **27** (Scheme 3) can be formed by condensation of PRA **13** and FAICAR **20** (N5‐formylAICAR) (Scheme 1, first and last step, respectively) and, then, by cyclization (**Scheme** **4**). ProFAR **28** and/or PrFAR **29** also could have been generated directly through this process that necessitates interactions and/or steric hindrance to prevent early intramolecular cyclization of FAICAR **20** to give IMP **21** (Scheme 2). Thereby, some cross‐reactions of the first metabolites selected for IMP biosynthesis may have participated to the emergence of the histidine biosynthetic pathway. Recently, ribosylamine was generated from glycolaldehyde, glyceraldehyde, and ammonia as an intermediate in a plausible prebiotic synthesis of NAD+.^[^ ^104^ ^]^ In this approach, the synthesis of the ribose ring and the synthesis of the nicotinamide heterocycle were combined to avoid the unselective glycosylation step also providing an interesting abiotic access to ribosylamine and, potentially, to PRA **13**, AICAR **10**, and IMP **21**. Future developments from this work could offer an answer to the question of how ribose has emerged in purine ribonucleotides (question discussed at the beginning of the introduction).

In the search for additional relationships between the purine ribotide and histidine pathways (see Part II Section Key pyrimidine ring‐opening in the histidine biosynthetic pathway and abiotic pyrimidine ring‐opening in N1‐modified adenine ribos(t)ides), two main chemical considerations can be underlined. First, the histidine biosynthesis is based on the key pyrimidine ring‐opening in PRAMP **27** that is a N1‐ribosyl AMP derivative (Scheme 3). Second, AICAR **10**, CAIR **18**, SAICAR **19,** and FAICAR **20** (Scheme 1) and/or analogs could result from IMP and/or AMP chemical modifications produced by pyrimidine ring‐opening reactions. Accordingly, in Part II, we review works on the chemistry of purine ribonucleos(t)ides and related purines, potentially performed under plausible prebiotic conditions, with a particular emphasis on ring‐opening reactions.

## 3 Part II. Abiotic Purine Chemistry, an Access to Imidazole and Pyrimidine Derivatives

In the de novo biosynthetic pathway of purine ribotides, five imidazole riboside 5’‐monophosphates were selected as intermediates (Scheme 3). The question of why the purine ring biosynthesis involves imidazole intermediates and not pyrimidine derivatives useful for complementary pyrimidine ribonucleotides and RNA biosynthesis merits attention.^[^ ^6^ ^]^

The proposed unified mechanism for abiotic synthesis of purine **1** and adenine **2** in formamide is reminiscent of the biosynthesis of purine nucleobases since it involves a critical glycine intermediate and imidazole derivatives according to the role of glycine in the biosynthesis of IMP (Scheme 1 (2nd step) and Figure 3).^[^ ^6^ ^]^ The key nucleobase in the biosynthesis of purine ribotides, hypoxanthine **4** also appeared to be formed from glycine, urea, and formic acid^[^ ^105^ ^]^ and, then, was converted to adenine **2** under plausible prebiotic conditions.^[^ ^106^ ^]^ Guanine and diaminopurine were also obtained through the condensation of *N*,*N*′‐diformylbiuret with glycinamide in the presence of P_2_O_5_.^[^ ^107^ ^]^

However, relationships with the corresponding abiotic and biosyntheses of the corresponding ribonucleos(t)ides need to be established. In this section, looking for additional interconnections, we review works on potentially useful abiotic synthesis routes to purines and purine ribonucleos(t)ides from pyrimidine derivatives, and, then, purine chemical modifications in ribonucleo(s)tides/RNA leading to imidazole and/or pyrimidine derivatives under plausible prebiotic conditions.

### 3.1 Abiotic Synthesis of Purines and Related Nucleic Bases, Nucleosides, and Nucleotides

**Scheme 5:**
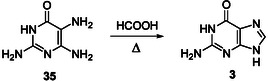
Last step of the Traube synthesis of guanine achieved from the pyrimidine **35** and formic acid.^[^ ^110^ ^]^

Chemists synthesized many purines from pyrimidine or imidazole derivatives, for example, in the search for new drugs.^[^ ^108^, ^109^ ^]^ The Traube purine synthesis using *ortho*‐diaminopyrimidine intermediates, such as **35** (**Scheme** **5**), is the most widely adopted route to prepare purines due to its great versatility.^[^ ^5^, ^110^ ^]^ This method has been described in 1900 with the synthesis of guanine **3** from guanidine. In the Traube synthesis of unsubstituted C8 purines, the very efficient one‐pot last steps of formylation/cyclization/water elimination have been initially made simply from formic acid (Scheme 5). Formamide may also replace formic acid^[^ ^111^ ^]^ and, urea and thiourea were employed to access to 8‐oxo and 8‐thiopurines. The abiotic synthesis of purines from imidazole derivatives is more rarely used, probably due to difficult access to various imidazole intermediates and the corresponding lack of versatility.^[^ ^108^, ^109^ ^]^

**Scheme 6:**
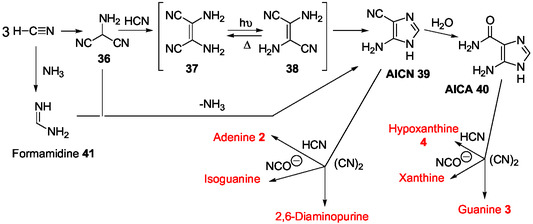
Abiotic synthesis of purines from hydrogen cyanide.^[^ ^1^ ^]^

Under plausible prebiotic conditions, pyrimidine and purine bases can be concomitantly formed from hydrogen cyanide and related derivatives,^[^ ^1^, ^6^ ^]^ for example, from formamide.^[^ ^1^, ^112^, ^113^ ^]^ The prebiotic synthesis of purine nucleic bases was mainly related to formation of intermediate imidazole derivatives such as 5‐aminoimidazole‐4‐carbonitrile AICN **39** ^[^ ^114^, ^115^ ^]^ and 5‐aminoimidazole‐4‐carboxamide AICA **40** from hydrogen cyanide and/or diverse derived reagents, cyanoacetylene, cyanoacetaldehyde, formamide, and urea (**Scheme** **6**).^[^ ^1^, ^4^, ^5^, ^6^, ^115^, ^116^, ^117^, ^118^, ^119^ ^]^ Formamidine **41** is an example of condensation product of hydrogen cyanide and ammonia involved in the adenine base (**2**) synthesis through intermediate formation of **39** (Scheme 6). AICN **39** is given by hydrolysis, AICA **40**, structurally related to AICAR **10**, that leads to the oxopurine bases, guanine **3** and hypoxanthine **4**. The reaction of AICN **39** with hydrogen cyanide also produces adenine. However, the encountered difficulty of ribose synthesis and nucleic base ribosylation makes unlikely a direct relationship between the prebiotic syntheses of nucleobases and corresponding ribos(t)ides^[^ ^1^ ^]^ and, therefore, between the abiotic synthesis of AICA **40** and that of the corresponding nucleoside and mononucleotide AICAR **10**.

**Scheme 7:**
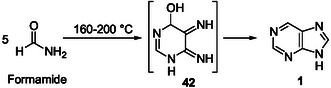
Abiotic synthesis of purine **1** from formamide through formation of the *o*‐diiminopyrimidine intermediate **42**.^[^ ^122^, ^123^, ^124^ ^]^

The prebiotic formation of purines via *o*‐diaminopyrimidines or related derivatives was more rarely reported than the corresponding synthesis via imidazole intermediates. Eschenmoser and Lowenthal reported the formation of tetra‐aminopyrimidine as a prebiotic intermediate to access to 2‐aminoadenine, riboflavin, and folic acids.^[^ ^120^, ^121^ ^]^ We may underline the inherent difficulty to demonstrate the formation of purines from *o*‐diaminopyrimidine intermediates since their reactions to give purine bicycles should be highly favored in the presence of the prebiotic reagents investigated (monocarbon reagents) according to the Traube purine synthesis. However, a related derivative, *o*‐diiminopyrimidine **42**, was identified as an intermediate in the synthesis of purine **1** from formamide solely heated at 160–200 °C without any other reagent (**Scheme** **7**).^[^ ^122^, ^123^, ^124^ ^]^ Experiments and calculations demonstrated that the corresponding reaction path without the involvement of aminoimidazole‐carbonitrile intermediates is also a viable alternative for the nonaqueous synthesis of nucleobases.^[^ ^125^ ^]^

**Scheme 8:**
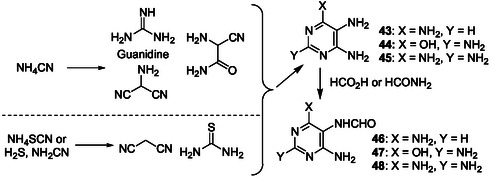
Production under plausible prebiotic conditions of diaminopyrimidines **43–45** and corresponding Fapy derivatives **46–48**.^[^ ^126^ ^]^

Recently, to generate purine ribonucleosides from new plausible prebiotic intermediates, diaminopyrimidines **43**–**45** were obtained from simple species such as NH_4_CN (**Scheme** **8**).^[^ ^126^ ^]^ Guanidine (available from cyanamide and NH_3_), for example, reacts, with the HCN trimer aminomalononitrile to produce tetra‐aminopyrimidine **45** (72%). **43–45** were selectively and simply formylated with formic acid or formamide to give, in high yields, the corresponding 6‐amino‐5‐formamidopyrimidines (Fapys) **46–48** (Scheme 8). **46–48** were also produced from hydrogen cyanide and nitrites through intermediate formation of 5‐nitrosopyrimidines.^[^ ^127^, ^128^ ^]^

**Scheme 9:**
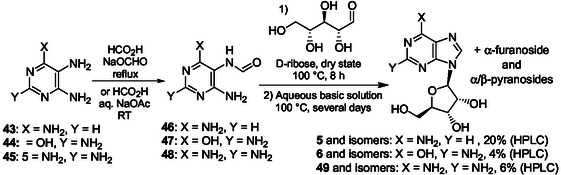
Proposed abiotic synthesis pathway to N9 adenine, guanine, and 2,6‐diaminopurine ribonucleosides **9**, **11**, and **49** formed through condensation of formamidopyrimidines **46**, **47**, and **48** with D‐ribose in the dry state, respectively.^[^ ^126^ ^]^

Remarkably, Fapy **46–48** heating with D‐ribose at 100 °C in the dry state followed by heating in aqueous solution under basic conditions for several days produce N9‐purine ribosides without formation of N7‐isomers through selective glycosylation (**Scheme** **9**).^[^ ^126^ ^]^ Thereby, adenosine **5**, guanosine **6**, and 2,6‐diaminopurine riboside **49** and the corresponding α‐ribofuranoside and α/β‐ribopyranosides were regioselectively produced. Remarkably, for example, adenosine was formed in 20% yield high pressure liquid chromatography (HPLC) using aqueous ammonia as a base. The highest total yield for N9‐ribosides of up to 60% was achieved using simple amine as bases.

This purine riboside formation pathway, named Fapy pathway, was fused to sugar‐forming reactions to produce ribosides to provide a plausible scenario of how purine nucleosides may have formed under prebiotic conditions.^[^ ^127^ ^]^ The Fapy chemical pathway was developed in a prebiotically plausible geochemical environment, driven solely by wet–dry cycles, without sophisticated isolation and purification procedures. It delivered both canonical and noncanonical purine nucleosides from ribose through formation of variously substituted 5‐nitrosopyrimidines that may be converted into Fapys in the presence of formic acid and elementary metals (Ni or Fe). A unified prebiotically plausible synthesis of pyrimidine ribonucleotides from small molecules and ribose, involving solely wet–dry cycles was shown compatible with the previously reported Fapy pathway giving purine ribonucleosides.^[^ ^128^ ^]^

These works demonstrate that *o*‐diaminopyrimidines 1) can be formed under plausible prebiotic conditions and can be intermediates in the synthesis of Fapys and the corresponding ribonucleosides; and, 2) allow from ribose, the selective formation of N9 purine ribonucleosides without production of N7‐isomers. Unnatural highly functionalized purine ribonucleosides were also synthesized in one pot through transient formation of *N,N*‐dimethylformamidino analogs of Fapys generated from *o*‐diaminopyrimidines and Vilsmeier reagents.^[^ ^129^, ^130^ ^]^

While strong progress has been made in the prebiotic synthesis of purine ribos(t)ides from purine bases or Fapys and ribose, the synthesis of ribose or related intermediates remains to be achieved more efficiently and more selectively. Therefore, the identified plausible prebiotic synthesis of purines and related ribos(t)ides cannot enlighten completely the choice of a purine ribos(t)ide biosynthesis proceeding through formation of imidazole intermediates such as AICAR. However, we may conclude that purines can be formed both from plausible prebiotic imidazole and pyrimidine intermediates. Accordingly, hereinafter, we examine the potential role of ring‐opening reactions of purine nucleobases, ribonucleotides, and RNA in the formation of histidine analogs and emergence of key metabolic pathways.

### 3.2 Abiotic Ring‐Opening Reactions of the Purine Bicycle in Nucleobases, Ribonucleos(t)ides, and Derivatives

In this section, two questions are addressed. First, is it possible, under plausible prebiotic conditions, to generate histidine analogs or related imidazole ribos(t)ides from purine nucleobases or corresponding ribonucleos(t)ides or from RNA? Second, can Fapys and/or *o*‐diaminopyrimidine derivatives be produced under plausible prebiotic conditions from purine bases and corresponding ribos(t)ides and from primitive RNA?

#### 3.2.1 Adenine and Guanine Decompose to Produce Imidazole Derivatives

**Scheme 10:**
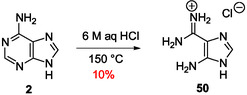
Pyrimidine ring‐opening in adenine under acidic conditions.^[^ ^131^ ^]^

Interestingly, under strong acidic conditions (6 m aqueous HCl in a sealed tube at 150 °C), adenine gives glycine and, in 10% yield, 5‐aminoimidazole‐4‐carbamidine **50** (**Scheme** **10**) structurally closely related to AICA structure **40**.^[^ ^131^ ^]^ Previously, 4 (or 5)‐guanidino imidazole has been isolated after subjecting guanine to nearly the same hydrolytic conditions.^[^ ^132^ ^]^ Therefore, in aqueous solution, under harsh acidic conditions, adenine and guanine decompose to give imidazole derivatives.

Adenine **2**, guanine **3**, and hypoxanthine **4** are stable under high alkaline aqueous conditions in the absence of another reagent. Abstraction of the more acidic hydrogen atom of guanine and hypoxanthine occurs in water under mild basic conditions due to the corresponding pK_a_ values lower than 10 and, thereby, corresponding N‐alkylation is possible under plausible prebiotic conditions in aqueous solution or at the dry state. Under basic conditions, N9‐alkylation happens mainly from adenine and guanine. Acylation is more difficult due to the potential hydrolysis of the acylation reagent and products but can proceed under phase transfer conditions or in the dry state after N9‐alkylation. However, only N6‐acylated‐9‐alkyladenine and N2‐acylated‐9‐alkylguanine derivatives and related nucleos(t)ides are significantly stable in the presence of water.

#### 3.2.2 N1‐ or N7‐Alkylation or Acylation Facilitates Ring‐Opening of the Purine Bicycle in Ribonucleos(t)ides and N9‐Alkylated Nucleic Bases

Whereas adenosine, guanosine, and inosine are stable under basic conditions in the absence of another reagents, N9*‐*ribofuranosyl purine (**1**), N9‐ribopyranosyl purine derivatives, and 9‐methylpurine were found to be extremely labile toward dilute aqueous alkali at room temperature (0.04 m aqueous NaOH).^[^ ^133^ ^]^ They give the corresponding Fapys and, then, in a second step, *o*‐diaminopyrimidines through cleavage of the formamido group. Therefore, if formed in the RNA world, such purine ribonucleos(t)ides probably have not been present in primitive RNAs and have been source of Fapy and diaminopyrimidines.

The lability of the purine bicycle was utilized by chemists to synthesize, from purines, numerous new imidazoles, pyrimidines, purines, and other heterocyclic derivatives.^[^ ^134^ ^]^ A selective modification/activation step of the imidazole or pyrimidine ring in less reactive purines was often introduced to facilitate the selective cleavage of one ring. The pyrimidine or the imidazole ring(s) of the purine bicyclic system was opened selectively and efficiently after N‐alkylation, N‐acylation, N‐glycosylation, N‐oxidation, N‐sulfonylation, and N‐nitration in the corresponding opened ring. A recent review article summarized these works,^[^ ^134^ ^]^ after a book chapter published in 1972.^[^ ^135^ ^]^ The pyrimidine ring‐opening of the purine bicycle, giving imidazole derivatives, is mainly favored by N1‐ and N3‐alkylation, N1‐nitration, or ‐ oxidation. The imidazole ring‐opening, leading to pyrimidines such as Fapys, is favored by N7‐alkylation or C8‐functionalization of N9‐alkyl derivatives such as purine nucleosides. Ring‐opening of nonactivated purines can also afford imidazole derivatives under stronger basic or acidic conditions.^[^ ^134^ ^]^ Thus, to facilitate the ring opening in hypoxanthine, adenine, and guanine ribonucleos(t)ides, N‐alkylation in aqueous solution under acidic, neutral or basic conditions is possible. Under acidic conditions, N‐alkylation competes with protonation. Under basic conditions, inosine and guanosine can be deprotonated to be alkylated mainly at N1. However, different monoalkylated products are formed and polyalkylation may take place. The position of alkylation is also dependent on the structure of the alkylating reagent.

#### 3.2.3 Pyrimidine Ring‐Opening in Purine Nucleobases and Purine Ribonucleos(t)ides

##### The Dimroth Rearrangement in N1‐Alkylated Adenine and Adenosine Derivatives, and in N6‐Acylated Adenine Derivatives

**Scheme 11:**
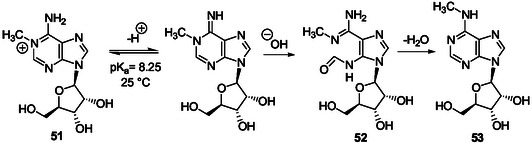
Dimroth rearrangement of 1‐methyladenosine under basic conditions.^[^ ^139^ ^]^

The Dimroth rearrangement is a translocation of two heteroatoms in a heterocyclic system, with or without changing the ring structure.^[^ ^136^ ^]^ In the purine series, this rearrangement concerns mainly adenine derivatives and results from the presence of the reactive adenine 6‐amino group. It is characteristic of the rich potential of the purine chemistry and occurs mainly under alkaline conditions through pyrimidine ring‐opening followed by ring closure to form a new purine derivative (for example, **Scheme** **11**). It consists in a nucleophilic addition of, mainly, hydroxide ion or water molecule on the electrophilic C2 atom inducing pyrimidine ring‐opening and formation of a formamidoimidazole (Faim) intermediate. Intramolecular ring‐closure in the Faim intermediate occurs through reaction of formamido and amino groups. Under stronger basic conditions, the formamido group of the Faim intermediate can be removed to give a 5‐amino imidazole derivative. Dimroth rearrangements were extensively reported for N9‐alkyladenine derivatives such as nucleos(t)ides modified at N1‐position. N6‐alkylated or N6‐acylated adenine derivatives also may undergo transformations through Dimroth rearrangements. The presence of a strongly electron‐withdrawing group at N1 facilitates the ring‐opening and the cleavage of the generated formamido group. For example, adenosine N1‐oxide derivatives were converted efficiently to imidazole derivatives.^[^ ^134^, ^137^ ^]^

*Dimroth Rearrangements in N1‐Alkylated Purine Ribonucleotides*

Methylation of adenosine **5** with dimethylsulfate, in DMF in the presence of potassium carbonate at 70 °C for 2 h, gives a mixture of 1‐methyl (**51**, Scheme 11), 3‐methyl, and 1,3‐dimethyl adenosine.^[^ ^138^ ^]^ These alkylated products were identified from the corresponding deglycosylated products isolated after hydrolysis in 1 m aqueous HCl at reflux for 1 h. Interestingly, 5‐aminoimidazole‐4‐*N*‐methylcarbamidine was also isolated as a product of hydrolysis. The Dimroth rearrangement of 1‐methyladenosine **51** happens at room temperature and at a rate proportional to the hydroxide ion concentration below pH 8 and above pH 10, with a plateau between, to give 6‐methyladenosine **53** through formation of the imidazole intermediate **52** (Scheme 11).^[^ ^139^ ^]^

**Scheme 12:**
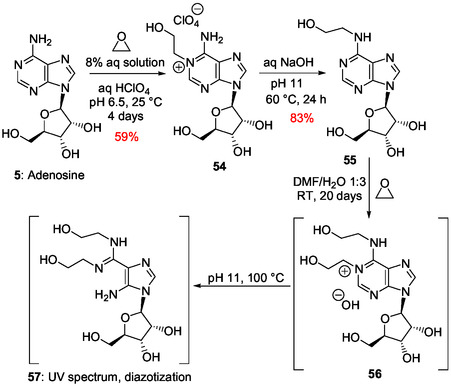
Monoalkylation of adenosine with ethylene oxide and corresponding Dimroth rearrangement followed by a second alkylation and pyrimidine ring‐opening under stronger basic conditions.^[^ ^140^ ^]^

The Dimroth rearrangement of 1‐methyladenosine brings out the high lability of the pyrimidine ring in N1‐alkyladenosine derivatives that may be formed from adenosine with various alkylating reagents in aqueous solution under mild basic conditions. The easy N1‐alkylation of adenosine and derivatives was demonstrated for adenosine **5**, ATP, and NAD+ through reaction with ethylene oxide in aqueous solution, at pH 6.5 or 6.0 and room temperature.^[^ ^140^ ^]^ For example, the N1‐hydroxyethyl derivative salt **54** (**Scheme** **12**) was slowly formed from adenosine (equilibrium at pH 6.5 after 70 h with addition of HClO_4_) and isolated in 59% yield (80% of the adenosine‐ethylene oxide reaction products from 14 g of adenosine). Thereby, adenosine N1‐alkylation is also possible efficiently in nearly neutral aqueous solution at room temperature. The corresponding Dimroth rearrangement was realized by heating **54** in aqueous NaOH at pH 11 and 60 °C for 24 h to give N6‐hydroxyethyl adenosine **55** in 83% yield (Scheme 12). The rearrangement also takes place under a large variety of pH, at pH 9 and 4 °C, for example.^[^ ^140^ ^]^ The 6‐monoalkyl product **55** also reacted with ethylene oxide to produce the 1,6‐dialkyl derivative **56** at room temperature in a DMF/water mixture (Scheme 12). Analysis by spectrophotometry of the alkaline solution of **56** heated at 100 °C suggested the formation of the imidazole riboside **57** resulting from the pyrimidine ring‐opening in **56** and cleavage of the generated formamido group. This study also brings out the possible formation of dialkylated imidazole ribonucleosides from adenosine under not too harsh basic conditions allowing pyrimidine ring‐opening and cleavage of the resulting formyl group.

*Dimroth Rearrangements Due to N‐Acylation in N6‐Glycinyl Adenine and Corresponding 9‐Methyl Derivative*

**Scheme 13:**
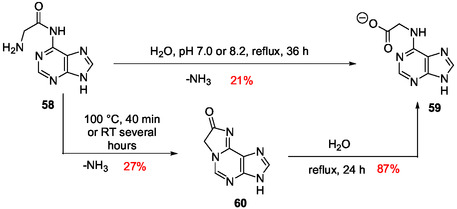
Rearrangement of 6‐glycinyladenine **58** in aqueous solution.^[^ ^141^ ^]^

Dimroth rearrangements resulting in a surprising chemical transformation in aqueous solution well illustrate the rich chemical diversity of products offered by purine reactions under plausible prebiotic conditions. Chheda and Hall synthesized 6‐glycinyladenine **58** and converted it to N6‐carboxymethyl adenine **59** in 21% yield simply by heating at reflux in aqueous solution (**Scheme** **13**).^[^ ^141^ ^]^ This transformation involved presumably a nucleophilic attack at C2 by a water molecule resulting in ring‐opening, elimination of ammonia followed by cyclization to give the tricyclic derivative **60**, hydrolyzed in the next steps. The tricyclic intermediate **60** was isolated and, then, synthesized to confirm the proposed mechanism. It was hydrolyzed in water at reflux and produced **59** in 87% yield (Scheme 12). Adenine, AICA **39**, and the corresponding carbamidine **40** were also detected as products, from **59** or **60** treated under neutral or basic conditions, in ratios depending on the conditions. The transient formation of the N1‐carboxymethyl isomer of **59** was also observed.

**Scheme 14:**
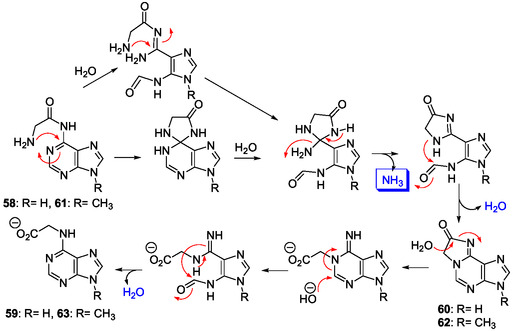
Mechanism of rearrangement of 6‐glycinyladenine derivatives **58** and **61** (R=H and R=CH_3_, respectively).^[^ ^143^ ^]^

A similar reactivity was observed with 6‐glycinyl‐9‐methyladenine **61**, analog of **58**, as model of the corresponding 6‐glycinyladenosine derivative (**Scheme** **14**). It gave **63** through formation of the tricyclic intermediate **62** that was also prepared.^[^ ^142^ ^]^ The mechanism of the corresponding complex rearrangement was investigated in details with ^15^N‐labeled compounds confirming the elimination of ammonia and/or ammonium ions and revealing its origin that is the N1 atom of the purine (Scheme 14).^[^ ^143^ ^]^

The rearrangements of the 6‐glycinyl adenine derivatives **58** and **61** are surprising and very interesting since they occur under plausible prebiotic conditions. Chheda and Hall reported that this behavior is probably general for all N6‐(α‐aminoacyl)adenines since they have isolated, from an aqueous solution of *N*6‐phenylalanyladenine, a cyclic product with properties corresponding to those of compound **60**.^[^ ^141^, ^143^ ^]^ The starting N6‐glycinyl‐adenine (**58**), N6‐glycinyl‐9‐methyl adenine (**61**), and the corresponding ribos(t)ides could be formed from glycine through water elimination, under plausible prebiotic conditions, by wet–dry cycles, for example. In aqueous solution, under plausible neutral or basic prebiotic conditions, N6‐acylated ribos(t)ide analogs of **59** and **63** could be formed through Dimroth rearrangements. The production of ammonia demonstrated from **58** and **61** (Scheme 14) might correspond to a general way of deamination under plausible prebiotic conditions of amino acids conjugated to adenine 6‐amino group. Moreover, an ammonia source is necessary in the IMP biosynthetic pathway, for conversion of PRPP **12** to PRA **13** and FGAR **15** to FGAM **16** (Scheme 1) and, in the histidine biosynthesis, for the conversion of PrFAR **29** to AICAR **10** and IGP **30**, and, conversion of IAP **31** to Hol‐P **32** (Scheme 3). The rearrangements of **58** and **61** also give numerous imidazole intermediates and products, such as AICA **39** and related carbamidines from **58**, and, their 9‐methyl derivatives from **61** (Scheme 14). Thereby, deamination of the corresponding adenosine and AMP glycinyl (and other α‐amino acyl) conjugates should produce nucleos(t)ides having structures closely related to those of AICAR **10**, SAICAR **19,** and FAICAR **20** (Scheme 1).

Formally, such a rearrangement can also be related to the biosynthetic purine pathway through adenylosuccinate **22** (6‐succinylAMP, Scheme 2) and 5‐formylated SAICAR (5‐formyl‐4‐succinylAICAR, Scheme 1). In the purine pathway, adenylosuccinate lyase (ASL) catalyzes the production of SAICAR **19** from CAIR **18** (Scheme 1) and the conversion of IMP **21** to adenylosuccinate **22** to biosynthesize adenosine (Scheme 2). Thereby, ASL is the only enzyme to catalyze two separate pathway reactions, enabling it to participate in the addition of the nitrogen N1 and N6 in AMP from aspartate.^[^ ^76^, ^77^, ^144^ ^]^

**Scheme 15:**
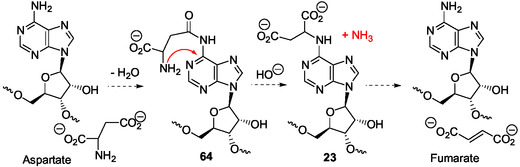
Proposed formation of succinyladenosine derivatives **23** from the condensation products **64** of aspartic acid and adenosine derivatives, according to a mechanism similar to the mechanism described for the rearrangement of 6‐glycinyladenine derivatives **58** and **61** (Scheme 14).

Probably, early under plausible prebiotic conditions, acylation of the N6‐amino group of adenine derivatives, such as adenosine, AMP, and adenine, free or in RNA, has occurred through water elimination in the dry state. For example, condensation with aspartic acid or aspartic anhydride derivatives^[^ ^49^ ^]^ can give **64** (**Scheme** **15**), analogs of the glycinyl derivatives **58** and **61**, resulting from the reaction of the less hindered and more reactive carboxylic acid function of aspartate with the 6‐amino group of adenine derivatives. Dimroth rearrangements of the ribonucleotide corresponding to **64** could produce the unstable ribonucleos(t)ides **23**, that correspond to adenylosuccinate **22** (Scheme 15) involved in the biosynthesis of AMP (Scheme 3), and, then, give fumarate (and maleate) under basic conditions and/or by heating with regeneration of the starting adenosine derivative **64**. Interestingly, in such a transformation, the last intermediate is the carbamidine analog of 5‐formyl SAICAR (Scheme 15). This latter and adenylosuccinate derivatives **23** also could be formed from isomers of **64** in which aspartic acid is conjugated by its α‐carboxyl group to adenosine 6‐amino group according to the mechanism established for formation of **59** and **63** (Scheme 14). Thus, the corresponding potential transformations merit attention and investigation from ribonucleos(t)ides **64** and isomers, and, for comparison, from 6‐glutamyl adenosine analogs.

##### Odd Bases in Transfer RNAs and Molecular Fossils: Adenosine N6‐Carbamoylation and Purine Ribonucleotide N‐Methylation

Since amino acid derivatives of adenine decompose to give imidazole derivatives structurally related to contemporary metabolites involved in the biosynthetic pathways of purine ribotides and of histidine, we review in this section some works highlighting the emergence of biochemical bonds between amino acids and purines.

**Figure 5:**
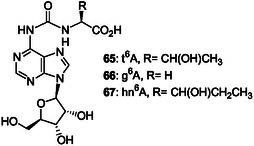
Odd nucleosides **65–67** identified in tRNA and resulting from conjugation of the 6‐amino group of adenosine to the amino group of α‐amino acids by a carbonyl group to form ureido derivatives.^[^ ^145^, ^146^, ^147^, ^148^, ^149^, ^150^ ^]^

Adenine bases carrying an amino acid group were identified as odd bases in tRNAs after enzymatic tRNA digestion. In their structure, a surprising stable carbonyl group links the 6‐adenine amino group to the amine function of threonine (t^6^A **65**),^[^ ^145^, ^146^, ^147^, ^148^ ^]^ glycine (g^6^A **66**),^[^ ^149^ ^]^ and 3‐hydroxynorvaline (hn^6^A **67**),^[^ ^150^ ^]^ to form very stable ureido nucleotides (**Figure** **5**).

**Scheme 16:**
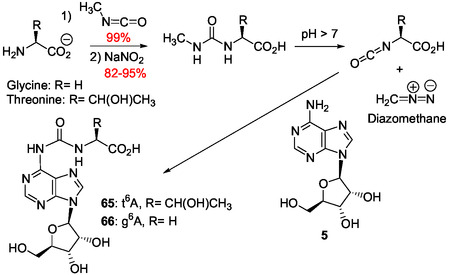
Formation under plausible prebiotic conditions of natural and synthetic ureido adenosine derivatives with production of diazomethane as a methylating agent.^[^ ^160^ ^]^

**Scheme 17:**
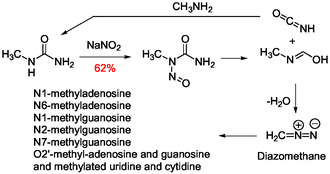
Nitrosylation of methylurea under plausible prebiotic conditions generating diazomethane able to methylate canonical tRNA bases.^[^ ^160^ ^]^

Many modified bases were identified in different tRNAs.^[^ ^40^, ^69^, ^70^, ^127^, ^151^, ^152^, ^153^, ^154^ ^]^ These odd bases result from post‐transcriptional enzymatic tRNA modifications^[^ ^70^, ^151^, ^152^, ^153^, ^154^ ^]^ inducing an optimal fit in aminoacyl‐tRNA/mRNA complexes into ribosome. Some of them are considered as molecular fossils of the RNA world.^[^ ^51^, ^52^, ^53^, ^54^, ^55^, ^60^, ^69^, ^70^, ^154^, ^155^, ^156^, ^157^, ^158^ ^]^ Seven tRNA mono‐modified canonical bases are amino acid derivatives and, four of them are purine derivatives, three N6‐ureido adenosine derivatives **65–67** (Figure 5), and the 7‐deazaguanosine derivative GluQ.^[^ ^154^ ^]^ Odd nucleosides **65–67** are found in the anticodon stem‐loop of tRNAs.^[^ ^69^, ^152^, ^154^ ^]^ Phylogenetic analyses and the fact that 6‐threoninyl adenosine (t^6^A) **66** (Figure 5) is found in all three kingdoms of life suggested that such amino acid‐modified bases were already present in the LUCA, from which all current life forms descended.^[^ ^60^, ^155^, ^156^, ^157^, ^158^, ^159^ ^]^ Recently, a plausible prebiotic route to the 6‐amino acid adenosine ureido conjugates found in tRNAs was reported with the synthesis of the glycine‐(g^6^A **65**) and threonine‐(t^6^A **66**) adenosines using isocyanates in combination with sodium nitrite to generate carbamoylating and methylating reagents under plausible prebiotic conditions (**Scheme** **16** and **17**).^[^ ^160^ ^]^ Carbamoylation of the corresponding amino acids was achieved in high yields using methylisocyanate. The majority of the methylated ribonucleosides identified in tRNAs as noncanonical bases^[^ ^161^, ^162^ ^]^ were also abiotically generated through nitrosylation of *N‐*methylurea with sodium nitrite to generate the methylating agent diazomethane (Scheme 17).^[^ ^160^ ^]^

A variety of natural and synthetic 6‐aminoacid adenosine derivatives made of an ureido link (valine, phenylalanine (L and D), serine, histidine, aspartic acid) were prepared and incorporated into oligoribonucleotides that were proposed as living molecular fossils of an extinct molecular RNA‐peptide world.^[^ ^163^ ^]^ From such a RNA–peptide world relics of the RNA world should have emerged ribosomal peptide synthesis through peptide synthesis directly performed on RNA.^[^ ^51^, ^52^, ^53^, ^54^, ^55^, ^56^, ^57^, ^58^, ^59^, ^60^, ^61^, ^62^ ^]^ Recently, first steps of peptide synthesis were achieved from g^6^A **65** tethered at the 5’‐extremity to a short oligoribonucleotide hybridized to an oligoribonucleotide carrying at its 3’‐extremity another tRNA odd nucleotide 5‐methylaminomethyluridine ((m)nm^5^U).^[^ ^60^ ^]^

The 6‐aminoacid‐modified adenosines **65–67** identified in different tRNAs are made of a surprising urea link. Probably, such a structure was selected in part for its high stability. For example, the threonine adenine conjugate **65** reported in 1964 is stable in 0.5 m aqueous HCl at 100 °C for 2 h, in alkaline buffer (pH 10.5) at room temperature for 24 h and in the presence of 2 M hydroxylamine (pH 7.0) at room temperature for 3 h.^[^ ^145^ ^]^ However, Dimroth rearrangements in **65–67** are possible. Other amino acids and other modes of conjugation to the base were present in the 7‐deazaguanine derivative GluQ and in odd tRNA pyrimidines (k^2^C, τm^5^s^2^U, and acp^3^U).^[^ ^154^ ^]^ The urea mode of conjugation of amino acids found in the odd purine bases preserves the anionic charge of the amino acid carboxylate, present at physiological pH, and, thus, add a repulsion effect regarding phosphodiester groups in the corresponding tRNA. Numerous other modes of amino acid attachment to purines have been probably “emerged” during the past life of RNAs, due to the chemical multifunctional characters of amino acids and purines. They have been not selected due to chemical instability, destabilizing or too highly stabilizing effects into different tRNA complexes. Therefore, N6‐acylation of adenosine and N1‐alkylation/acylation in free ribonucleos(t)ides and/or in RNA appear to be potential modes of activation of the purine bicycle, under plausible prebiotic conditions, to produce imidazole derivatives through Dimroth rearrangements. Such reactions of adenosine should have served a role in the development of a primary metabolism from damaged ribonucleotides and/or damaged RNA.

##### Pyrimidine Ring‐Opening in Inosine and IMP

In the search for ring‐opening reactions giving imidazole derivatives under plausible prebiotic conditions, the chemistry of hypoxanthine ribonucleos(t)ide derivatives (inosine and IMP) appears to be very attractive to investigate since adenine and guanine ribonucleotides (AMP and GMP) are synthesized in vivo from the corresponding inosine ribonucleotide IMP (Scheme 2). The pyrimidine ring of N1‐alkyl hypoxanthine and inosine derivatives is more easily opened than the corresponding ring in adenosine and guanosine derivatives due to the strong electron‐withdrawing effect of the carbonyl group (C6) and the absence of electron‐rich group at C2. After pyrimidine ring‐opening, the ring‐closure from O6‐atom to produce a Dimroth product is also not preponderant. N1‐nitro and N1‐(4‐nitrophenyl) inosines can also undergo ring‐opening through attack of amines at C2, followed by ring closure to reform the purine ring system with loss of the original N1 functionality.^[^ ^164^, ^165^ ^]^ Interestingly, the pyrimidine ring‐opening and ring‐closure reactions in uridines and inosines were used for the introduction of ^15^N labels at N3 of uridine and N1 of inosine derivatives using ^15^NH_3_ as a nucleophile to open the corresponding N‐nitro derivatives.^[^ ^164^ ^]^ Using alkylamines as nucleophiles, N1‐alkylinosine and N3‐alkyluridine derivatives were also prepared. Such reactions of uridine derivatives could be related to the nonenzymatic formation, from aminoethylglycine, of the tRNA odd base 3‐amino‐3‐carboxypropyluracil (acp^3^U). In *E. coli* tRNA, the acp^3^U base^[^ ^154^ ^]^ is produced by an aminocarboxypropyl (acp) transferase that was recently identified.^[^ ^166^ ^]^ Many examples of pyrimidine ring‐opening in hypoxanthine and inosine derivatives giving imidazole heterocycles have been reported and reviewed.^[^ ^134^ ^]^

**Scheme 18:**
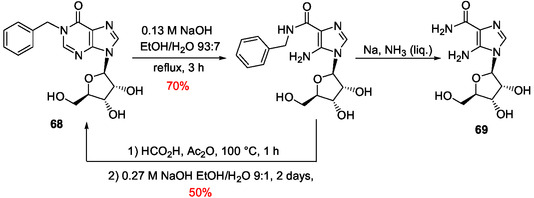
Synthesis of AICAR precursor **69** (ribosylated AICA **40**) from N1‐benzyladenosine **68**.^[^ ^168^ ^]^

Hereinafter, few representative examples of such ring‐openings, possibly achievable under plausible prebiotic conditions, at least in part, are summarized. The possible abstraction of the N1H of inosine (pK_a_ at 25 °C = 8.8)^[^ ^167^ ^]^ and 9‐alkylhypoxanthine derivatives by a mineral base (Na_2_CO_3_, Na_2_HPO_4_) allows N1‐alkylation in water or at the dry state, under plausible prebiotic conditions. Consequently, the pyrimidine ring in N1‐alkyl inosine derivatives can be opened by nucleophiles such as a water molecule and/or a hydroxide ion under mild basic conditions, to avoid from inosine the need for harsh conditions since “mild” deprotonation is possible.^[^ ^168^ ^]^ In his pioneer work in the field to prepare AICAR derivatives from inosine **7**, E. Shaw used alkaline conditions to retain the glycosidic link intact since the glycosidic bond of nucleosides is not stable under acidic conditions. This work was developed a little after the discovery of imidazole ribotides as intermediates in the biosynthesis of purine ribotides. In a first approach, the purine bicycle has been made labile to alkaline hydrolysis by benzylation of inosine at N1‐position.^[^ ^168^ ^]^ 1‐benzylinosine **68** has been hydrolyzed to give 5‐amino‐4‐imidazole‐*N*‐benzylcarboxamide riboside by expulsion of carbon atom 2 of the purine ring at reflux of a diluted NaOH solution in an ethanol/water mixture (**Scheme** **18**). Debenzylation of the imidazole nucleoside product by sodium in liquid ammonia gave the desired AICAR‐related nucleoside **69**.

**Scheme 19:**
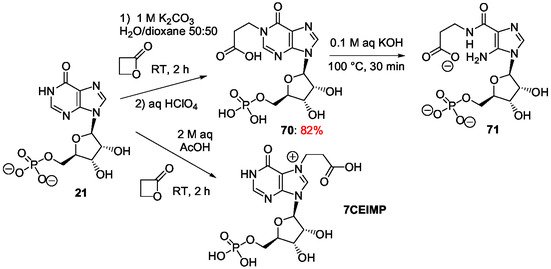
Synthesis of AICAR analog **71** from IMP **21**.^[^ ^169^ ^]^

In 1965, Baugh and Shaw reported a new approach in the synthesis of AICAR analogs under conditions more closely related to plausible prebiotic conditions.^[^ ^169^ ^]^ Under carefully controlled conditions of pH, the site of alkylation of IMP **21** by β‐propiolactone in aqueous solution was directed to provide in good yield either N1‐ or N7‐(2‐carboxyethyl)inosinic acid (1‐CEIMP **70** or its 7‐CEIMP isomer). Under neutral conditions, reaction at N7 of IMP was not taking place. However, at a lower pH, in 2 m aqueous acetic acid, 7‐CEIMP (**Scheme** **19**) was obtained whereas 1‐CEIMP **70** was formed and isolated under mild basic conditions (Scheme 19). The opening of the pyrimidine ring of **70** in aqueous 0.1 m KOH at reflux for 30 min gives *N*‐(5‐amino‐1‐β‐D‐ribofuranosyl‐imidazole‐4‐carbonyl)‐β‐alanine 5′‐phosphate **71** (Scheme 19), an analog of SAICAR **19** that is an intermediate in the biosynthetic pathway of IMP (Scheme 1). Extreme lability of 7‐CEIMP was observed in 1 m aqueous NaOH at RT for 30 min with the formation of an uncharacterized product that is probably the pyrimidine resulting from the opening of the imidazole ring as proposed by Baugh and Shaw. The easy and efficient N1‐alkylation of IMP in aqueous basic solution by β–propiolactone (82% yield at room temperature) to give a SAICAR analog suggests that lactones are efficient electrophilic species useful for N1‐alkylation of IMP in aqueous solution under plausible prebiotic conditions.

Various imidazole and AICAR derivatives were also prepared using different nucleophiles.^[^ ^134^ ^]^ For example, the introduction of N1‐nitroaryl groups in 2’,3’‐di‐*O*‐acetyl‐2′‐deoxyinosine strongly facilitates ring‐opening by ammonia, alkyldiamines, hydrazine, and hydroxylamine to give N1‐alkylated 2′‐deoxyinosine derivatives.^[^ ^165^ ^]^

Clearly, the work of Baugh and Shaw demonstrates that N1‐alkylation of hypoxanthine derivatives, inosine, and IMP, and the corresponding pyrimidine ring‐opening, are possible, under plausible prebiotic conditions, in neutral or basic aqueous solution, to generate imidazole derivatives that are SAICAR analogs. Such chemical reactions of purine ribos(t)ides, free or in RNA might have played a major role in the development of primary purine ribotide and histidine (bio)synthetic pathways.

##### Imidazole Ring‐Opening of the Purine Bicycle in N7‐Alkylated Ribonucleotides and Deglycosylation

**Scheme 20:**
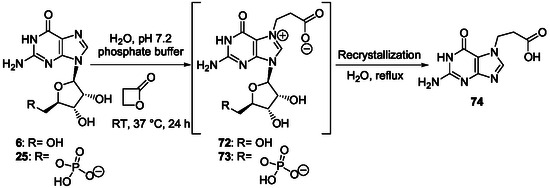
N7‐alkylation of guanosine **6** and GMP **25** with β–propiolactone and related deglycosylation.^[^ ^170^ ^]^

To investigate the mutagenic effect of β‐propiolactone, recently evidenced at this time, and in parallel to the work of Baugh and Shaw, Roberts and Warwick studied the reactions of guanosine **6**, guanosine 5’‐monophosphate **26,** and yeast RNA with the lactone in aqueous solution at pH 7.2.^[^ ^170^ ^]^ In each case, the main isolated product was 7‐(2‐carboxyethyl)guanine **74** (**Scheme** **20**) produced by 1 h heating at 100 °C in 1 m aqueous HCl of the final reaction mixture The positive charge carried by the formed N7‐alkyl guanosine derivatives **72** and **73** facilitates the deglycosylation through formation of the reactive oxocarbenium intermediate.

**Scheme 21:**
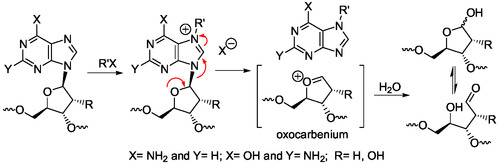
Mechanism of abasic site formation in DNA or RNA due to adenine and guanine N7‐alkylation and through formation of a reactive oxocarbenium intermediate.

These different works showed that alkylation of inosine **7**, IMP **21**, guanosine **6**, GMP **25**, and RNA is possible in aqueous solution under plausible prebiotic conditions. Under mild basic conditions, in aqueous potassium carbonate solution, inosine and IMP are mainly alkylated at N1, and, in acidic or neutral conditions IMP, guanosine, and GMP are alkylated at N7. The work of Roberts and Warwick also demonstrated the instability of N7‐guanosine derivatives leading to the formation N7‐alkyl guanines by deglycosylation under acidic conditions. Such a deglycosylation also arises with N3‐ and N7‐alkyladenosine derivatives, for example, from epichlorohydrin adenosine N7‐adducts.^[^ ^171^ ^]^ However, deglycosylation is more difficult for N7‐alkyl purine ribonucleos(t)ides, free or in RNA, in comparison to their DNA analogs due to the presence of the 2’‐OH group which destabilizes the oxocarbenium intermediate that is immediately trapped by water (**Scheme** **21**). For example, the apparent first‐order rate constant for acid hydrolysis of 2′‐deoxyadenosine is decreased by a factor of thousand in adenosine, under the same conditions, in 0.1 m aqueous HCl at 80 °C.^[^ ^66^ ^]^

**Scheme 22:**
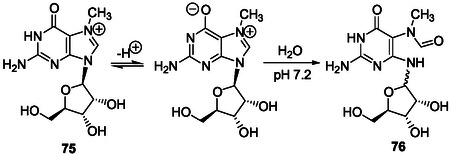
Imidazolium ring‐opening in 7‐methylguanosine giving Fapy derivative **76** under plausible prebiotic conditions.^[^ ^174^ ^]^

The formation of reactive apurinic (abasic sites) in DNA results from a simple adenine or guanine protonation or from N7‐alkylation by anticancer drugs, for example. Introduction of C8 electron‐withdrawing substituents in nonalkylated nucleosides also increases the rate of deglycosylation.^[^ ^172^, ^173^ ^]^ In N7‐alkylated adenosine and guanosine derivatives, deglycosylation competes with the imidazole ring‐opening giving Fapys under neutral or basic conditions. For example, deglycosylation at physiological pH 7.2 in 7‐methylguanosine **75**, that is a part of the cap allowing nucleus output of mature mRNA, appeared to be much slower than the imidazole ring‐opening and gives only **76** (**Scheme** **22**, *t*_1/2_ (**75**) 85 h at 25 °C).^[^ ^174^ ^]^

In contemporary DNA and RNA, formamidopyrimidine (Fapy) lesions are formed in competition with 8‐oxopurines from oxidative stress.^[^ ^175^ ^]^ In DNA, N7‐alkylation with anticancer drugs, for example, produces N‐alkylformamidopyrimidines. Little is known concerning the formation of N‐alkylFapys in RNA, perhaps due to their instability and/or the stability of the corresponding purinium precursor (Scheme 21). Most likely also, the lack of studies is related to DNA primacy as a molecular target of mutagenic or anticancer alkylating agents. From the reaction of yeast RNA with β‐propiolactone, for example, Roberts and Warwick isolated after acidic RNA hydrolysis the 7‐(2‐carboxyethyl)guanine **74** (Scheme 20) demonstrating RNA depurination from produced guanine imidazolium ions **72** and **73**.^[^ ^170^ ^]^ However, in the hypoxanthine series, the work of Baugh and Shaw suggests the formation of N‐alkylformamidopyrimidines through 7‐alkylation of IMP with β‐propiolactone producing N7‐(2‐carboxyethyl)IMP (7CEIMP, Scheme 19) which appeared to be extremely labile under basic conditions.^[^ ^169^ ^]^

In primeval RNA, Fapy damages resulting from C8‐oxidation and N7‐alkylation have been probably produced and source of further purine chemical modifications, strongly magnifying the molecular diversity suitable for a primary metabolism development (see next Section Imidazole ring‐opening in guanine ribonucleotides, formation of formamidopyrimidine (Fapy) intermediates).

#### 3.2.4 Metabolic Pathways Based on Ring‐Opening of Purine Ribonucleotides

##### Imidazole Ring‐Opening in Guanine Ribonucleotides, Formation of Formamidopyrimidine Intermediates

**Figure 6:**
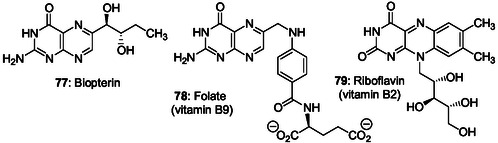
Structures of biopterin and vitamins biosynthesized from guanosine 5′‐triphosphate (GTP **80**, Scheme 23).

**Scheme 23:**
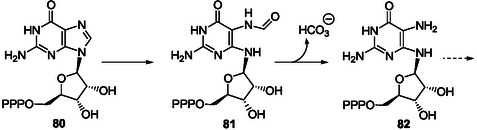
Opening of the imidazole ring of GTP **80** and decarboxylation performed by the enzymes GTP cyclohydrolases.^[^ ^176^, ^177^ ^]^

Different metabolic pathways probably took advantage of the rich nucleic base chemistry developed in RNA and/or from free purine ribonucleos(t)ides. The biosynthetic pathways of biopterin, folate (vitamin B9 **78**, **Figure** **6**), and, riboflavin (vitamin B2 **79**) and 5‐deazaflavin derivatives, and related cofactors (FMN and FAD, cofactor F420, etc.), are based on the ring‐opening of guanine ribonucleotides. These vitamins and cofactors are biosynthesized from GTP **80** (**Scheme** **23**) by the enzymes GTP cyclohydrolases via formation of Fapy ribonucleotides such as **81** ^[^ ^176^, ^177^ ^]^ formed through the nucleophilic attack of a water molecule at C8 of the imidazole ring (Scheme 23). In a second step, triaminopyrimidines **82** are formed as intermediates. The biosynthesis of some tRNA odd bases that are guanine derivatives (archaeosine and queuosine) is also catalyzed by cyclohydrolases.^[^ ^178^, ^179^, ^180^ ^]^ The corresponding biosynthetic pathways may be related to the chemistry of N7‐alkylated (or N7‐acylated) and/or C8‐oxidized guanosine derivatives.

Formation of Fapy **76**, by hydrolysis of 7‐methylguanosine **75** at physiological pH (Scheme 22),^[^ ^174^ ^]^ suggests that Fapy guanosine derivatives were probably formed under prebiotic conditions. Thereby, the produced Fapys can be precursors of vitamins and enzymatic cofactors and, initiators in the emergence of corresponding biosynthetic pathways involving GTP cyclohydrolases that could be vestiges of RNA repair enzymes.

**Scheme 24:**
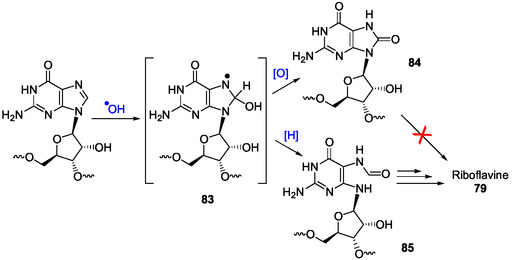
Proposed formation of Fapy nucleos(t)ides **85** as a product of reaction of guanosine and related ribonucleotides with a hydroxyl radical as a route toward riboflavine **79** (Figure 6) and the corresponding metabolic pathway involving GTP cyclohydrolase ([O]: one‐electron oxidization, [H]: one‐electron reduction).^[^ ^181^ ^]^

In the evolution of life, the important question of how UV‐induced photodamages have been repaired in primitive RNA, was discussed in a review article from the corresponding works.^[^ ^181^, ^182^, ^183^ ^]^ C. Burrows et al. highlighted the crucial role of formation of the oxidative damage product 8‐oxoguanosine (8‐oxo‐7,8‐dihydroguanosine) **84** (**Scheme** **24**) in the emergence of the enzymatic base repair using riboflavin‐based enzymatic cofactor. This damage was shown to mimic, in photorepair, the function of the isoalloxazine tricycle found in riboflavin **79** and related cofactors, FMN and FAD (Figure 6).^[^ ^182^ ^]^ Cyclobutane pyrimidine dimers are DNA damages formed by UV exposure that can be formed in RNA in vivo and under abiotic conditions. The corresponding DNA damages are repaired by the old enzymes photolyases present in bacteria, fungi, yeast, plants, and animals (not in man) using riboflavin‐related cofactors. When incorporated into a DNA or RNA strand in proximity to a cyclobutane pyrimidine dimer, 8‐oxoguanine nucleotide acts catalytically in a mechanism consistent with that of photolyase in which the photoexcited state of the oxopurine donates an electron to a pyrimidine dimer to initiate cyclobutane bond cleavage and, subsequent back electron transfer regenerates 8‐oxoguanosine.^[^ ^182^, ^183^ ^]^ In 8‐oxoguanosine derivatives **84**, the urea group formed from the guanine imidazole ring is difficult to hydrolyze to open the imidazole ring. A very interesting route toward the emergence of riboflavin **79** was proposed (Scheme 24) since enzymatic cofactors made of riboflavin, FMN and FAD, are involved in the enzymatic photorepair of cyclobutane pyrimidine dimer lesions.^[^ ^181^ ^]^ It consists in the formation of the intermediate radical adduct **83** through reaction of guanosine (as a nucleoside, nucleotide or in RNA) with hydroxyl radical and, then, reduction to give Fapy derivatives **85** related to **81** (Scheme 24) that is intermediate in the biosynthesis of the riboflavin **79** and related enzymatic cofactors (FMN, FAD, F412) (Scheme 24 and Figure 6). The radical intermediate **83** is given by oxidation the transient repair agent 8‐oxoguanosine **84**.^[^ ^181^ ^]^ More recently, a UV‐driven self‐repair of cyclobutane pyrimidine dimers also appeared possible.^[^ ^184^ ^]^

##### Key Pyrimidine Ring‐Opening in the Histidine Biosynthetic Pathway and Abiotic Pyrimidine Ring‐Opening in N1‐Modified Adenine Ribos(t)ides

The summarized works suggest that inosine, IMP, adenosine, and related nucleotides, free or in RNA, are suitable candidates as precursors, under prebiotic conditions, of imidazole ribotides such as AICAR through alkylation or acylation and pyrimidine ring‐opening. Indeed, the pyrimidine rings of N1‐alkylated inosine and adenosine and, N6‐glycinyl adenine derivatives, are opened in aqueous solution under neutral or mild basic conditions to give the corresponding imidazole derivatives. The emerging metabolic pathways producing purine ribotides can be seen as resulting from the instability of prebiotic purine ribos(t)ides derivatives. Like observed with purine ribos(t)ides, AICAR and analogs can be deglycosylated under acidic conditions to release AICA **40** made of an imidazole ring able of proton transfer for catalysis offering an access to histidine derivatives. Nevertheless, deglycosylation of AICAR derivatives was not selected as a step of the histidine biosynthesis, probably, by lack of selectivity in regard to the possible ribonucleotide deglycosylation.

In the first step of histidine biosynthesis (Scheme 2), ATP **11** is ribosylated at N1 by PRPP **12** to give 1‐phosphoribosylated ATP (PRATP **26**) that is dephosphorylated to provide PRAMP **27**.^[^ ^99^, ^185^ ^]^ Ribosylation at N1 of purine ribonucleos(t)ides favors nonenzymatic pyrimidine ring‐opening under basic conditions and corresponding Dimroth rearrangement.

**Scheme 25:**
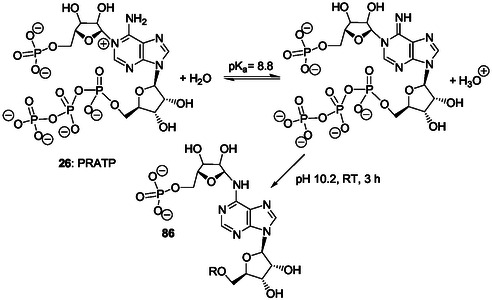
Protonation equilibrium of 1‐phosphoribosyladenosine 5’‐triphosphate PRATP **26**, intermediate in the biosynthetic pathway of histidine, and, corresponding Dimroth rearrangement observed by spectrophotometry in basic aqueous solution (undetermined R substituent, the triphosphate group was probably hydrolyzed to give the monophosphate).^[^ ^185^ ^]^

The key step allowing production of IGP **31** and AICAR **10** from PrFAR **30** involves pyrimidine ring‐opening in PRAMP through cleavage of the N1‐C6 purine bond. PRAMP is converted to ProFAR **29** by HisI that is a cyclohydrolase using magnesium and zinc ions.^[^ ^186^ ^]^ A mechanism involving a Zn^2+^‐mediated activation of a water molecule and a histidine residue as a general catalytic base was proposed that has features similar to, but distinct, from those of previously characterized purine and pyrimidine deaminases. In the Dimroth rearrangement of N1‐alkyladenosine derivatives leading to the corresponding N6‐alkylated derivatives, the N1—C2 bond is cleaved under neutral or basic conditions and, then, the pyrimidine ring is reformed. A first report on the stability of PRATP **26** suggested that this compound underwent the Dimroth rearrangement to form corresponding N6‐phosphoribosyl products **86** under basic conditions (**Scheme** **25**).^[^ ^99^, ^185^ ^]^ The evolution of the UV spectra in buffered aqueous solution, at pH 10.2 and room temperature during about 3 h appeared to be similar to that of 1‐methyl AMP converted to 6‐methyl AMP.^[^ ^185^ ^]^ This Dimroth rearrangement was also reported at pH 10 from optical rotation measurements.^[^ ^187^ ^]^ Interestingly, 8.8 pK_a_ value was assigned at 25 °C to the basic purine N1/N6 imine function of PRATP^[^ ^185^ ^]^ and, therefore, a large part of the corresponding species present at physiological pH are protonated on the pyrimidine ring of the purine bicycle (Scheme 25).

**Scheme 26:**
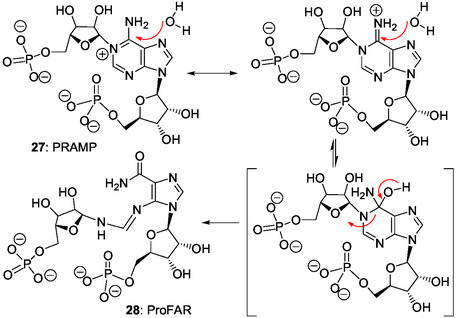
Mesomeric effect in PRAMP **27** protonated at physiological pH and related pyrimidine ring‐opening by nucleophilic attack of a water molecule at C6 to give ProFAR **28**.

The presence of a positive charge in PRAMP **27** at physiological pH due to N1‐ribosylation is probably at the origin of pyrimidine ring‐opening through a nucleophilic attack of a water molecule at C6 (and not at N2) to give ProFAR **28** (**Scheme** **26**). For comparison, in adenosine, N1 atom is the most basic with pK_a_ value of 3.34 at 25 °C.^[^ ^188^ ^]^ Such a ring‐opening reaction of N1‐alkyl adenosine derivatives could be possible under abiotic neutral conditions in the presence of zinc ions, for example.

Unfortunately, experimental data are lacking about hydrolysis and pyrimidine opening of PRATP **26** and PRAMP **27** under abiotic slightly acidic and neutral conditions. The reactions of N1‐alkyl adenosine derivatives such as 1‐methyladenosine, present in tRNA and rRNA, were also essentially studied under basic conditions in the perspective of their selective Dimroth rearrangement or under strong acidic conditions inducing deglycosylation. The hydrolysis of 1‐methyladenosine in 0.5 M aqueous HCl solution in a steam bath (80 °C) for 45 min gives, relatively rapidly, 1‐methyladenine, slowly transformed to 5‐aminoimidazole‐4‐*N*′‐methylcarbamidine.^[^ ^66^ ^]^ Under acidic conditions, the high degree of protonation due to the strong basicity of the 1,6‐imine group (pK_a_ = 8.25 at 25 °C)^[^ ^139^ ^]^ favors deglycosylation. Thereby, the abiotic hydrolysis of N1‐alkyl adenosine derivatives remains to be studied under neutral or slightly acidic conditions that may favor the cleavage of the N1‐C6 bond in water.

**Scheme 27:**
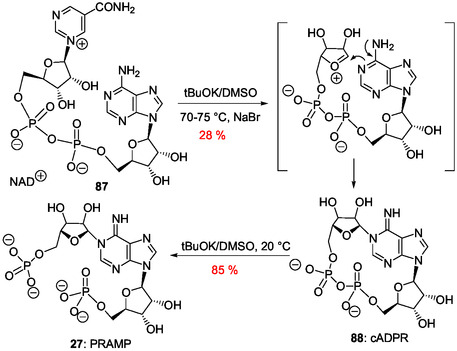
Formation of cADPR by heating of NAD+ with potassium *t*‐butoxide in the presence of sodium bromide in DMSO.^[^ ^190^ ^]^

Cyclic ADP‐ribose (cADPR **88**, **Scheme** **27**) is an interesting example of N1‐ribosylated adenosine derivative parent of PRATP and PRAMP. It is a second messenger strictly involved in the homeostasis of cellular calcium ions. cADPR is biosynthesized from NAD(P)+ **87** and can be simply formed in 28% yield by NAD+ heating under anhydrous conditions in the presence of sodium bromide and, of a strong base, potassium tert‐butoxide (t‐BuOK) (Scheme 27).^[^ ^189^, ^190^ ^]^ Interestingly, at room temperature, in DMSO in the presence of tBuOK, cADPR gives in high yield PRAMP **27** (Scheme 3 and 27).^[^ ^190^ ^]^ Recently, NAD+ **87** was synthesized under plausible prebiotic conditions from ammonia, cyanoacetaldehyde, prop‐2‐ynal, and sugar‐forming precursors, yielding in situ the nicotinamide riboside.^[^ ^104^ ^]^ Regioselective phosphorylation to form the 5’‐monophosphate and, then, condensation with water‐stable light‐activated adenosine phosphoramidate derivatives gave NAD+.

**Scheme 28:**
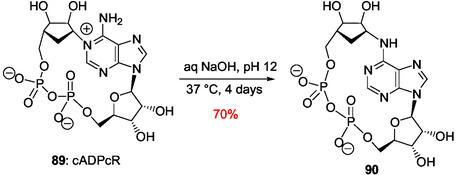
Structure of the analog of cADPR, cADPcR **89**, in which the 1‐phosphoribosyl core is replaced by a carbocyclic‐ribose and structure of the corresponding Dimroth‐rearrangement product.^[^ ^192^ ^]^

Jacobson et al. reported that alkaline treatment of cADPR in water does not result in a Dimroth rearrangement but instead in slow hydrolysis at the N1‐position to afford adenosine diphosphate‐ribose (ADP‐ribose) because of its unstable N1‐glycosidic linkage.^[^ ^191^ ^]^ A stable analog of cADPR, cADPcR **89**, in which the 1‐phosphoribosyl core is replaced by carbocyclic‐ribose was synthesized, and, its stability under acidic, neutral, and basic conditions was studied in comparison to cADPR (**Scheme** **28**).^[^ ^192^ ^]^ cADPcR, unlike cADPR, was relatively stable under neutral and acidic conditions, where under basic conditions, it formed in good yield the Dimroth‐rearranged N6‐cyclized product **90** (aqueous NaOH, pH 12, 37 °C, 4 days, 70% yield) (Scheme 28). cADPR gives under these conditions at least three products that were not identified. Decomposition of cADPR under acidic and neutral conditions produces ADP‐ribose identified by HPLC (*t*_1/2_ of 34 h at pH 2.0 and 60.5 h at pH 7.0). Therefore, 1‐ribosyladenosine derivatives appear to be unstable under acidic, neutral, and basic conditions.^[^ ^192^ ^]^

According to the reported results, PRAMP **27** involved in the biosynthesis of histidine gives, under basic conditions, the corresponding N6‐ribosyl adenine ribonucleotides through Dimroth rearrangement. PRAM hydrolysis and corresponding pyrimidine ring‐opening to form ProFAR **28** should be performed under very mild conditions at a pH close to physiological pH.

**Scheme 29:**
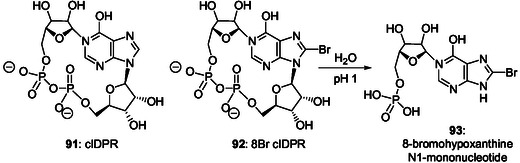
Structure of the inosine analog of cADPR, cIDPR **91**, and hydrolysis in acidic aqueous solution of 8‐bromo cIDPR derivative **92** to give nucleotide **93** of a novel class.^[^ ^195^ ^]^

**Scheme 30:**
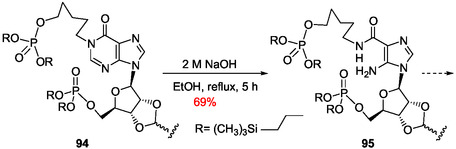
Step of pyrimidine ring‐opening in the solid‐phase synthesis of a new AICAR derivative from inosine.^[^ ^196^ ^]^

Matsuda et al. were the first who synthesize analogs of cADPR such as cIDPR **91** (**Scheme** **29**), in which hypoxanthine bicycle replaces adenine base.^[^ ^193^ ^]^ Various other inosine analogs of cADPR, stable under hydrolytic physiological conditions, were synthesized as tools for biological studies.^[^ ^193^, ^194^, ^195^, ^196^ ^]^ An unusual cleavage at pH 1 of one of them, cyclic 8‐bromoinosine diphosphate ribose **92**, was reported to give, by N9‐ribosyl scission and subsequent pyrophosphate cleavage, 8‐bromo‐*N*1‐ribosyl hypoxanthine 5’‐monophosphate **93**, a novel class of mononucleotide, as the sole product (Scheme 29).^[^ ^195^ ^]^ Under basic conditions, for example, a AICAR‐based cADPR mimic was prepared by solid‐phase synthesis involving pyrimidine ring‐opening in **94** to give the intermediate **95** (**Scheme** **30**) and cyclization through pyrophosphate bond formation.^[^ ^196^ ^]^

To the best of our knowledge, abiotic cleavage of the N1‐C6 purine bond in N1‐alkyladenosine derivatives was not reported. However, such a hydrolytic cleavage is probably possible under abiotic neutral or slightly acidic conditions, in the presence of zinc ions, for example. Due to ribosylation at N1, the imino group formed (pK_a_ at 25 °C = 8.8) is protonated at N6 in a large part of the PRAMP species present at physiological pH (Scheme 25 and 26). The resulting positive charge favors hydrolysis to produce ProFAR (Scheme 25) and, thus, N1‐ribosylation or N1‐alkylation is necessary to break by hydrolysis the N1‐C6 adenosine bond under neutral conditions like performed by cyclohydrolase HisI to generate ProFAR.^[^ ^186^ ^]^

The works, summarized in this section, highlight the key role of N‐alkylation and N‐acylation reactions in the purine chemical reactivity inducing different modes of pyrimidine ring‐opening from N1‐alkylated and N6‐acylated adenine derivatives, under abiotic and/or biological conditions. Thereby, under prebiotic conditions, many imidazole derivatives, structurally related to metabolites involved in the purine ribotide and histidine pathways, would have been produced from purine ribonucleotides and RNA.

## 4 Part III. Prebiotic Catalysis by Adenine and N6‐Modified Adenine Derivatives

Histidine has a central role in biochemical catalysis and as a ligand for metallic ion complexation in heme and nonheme enzymes and carrier proteins. AICA **40** (Scheme 5), formed under prebiotic conditions from hydrogen cyanide, and, related small reagents, can have played a role in catalysis and as metallic ion ligand under prebiotic conditions. However, the accumulation of AICA, for ribosylation under prebiotic conditions to give AICAR **10** (Scheme 1 and 3), in media containing reactive compounds, is unlikely due to the reactivity of the imidazole ring and of its substituents to give various purines (Scheme 6).

Adenine is an amphoteric molecule (pK_a_s 4.2 and 9.8 at 25 °C) which can be a relay for double proton transfer and catalysis in an early RNA world like imidazole derivatives or histidine at a different pH range. Several tautomeric equilibria in adenine favor such a proton transfer, potentially possible from five nitrogen atoms, and, therefore, advantageously in comparison to histidine, in different directions. A review article published in 2010 pointed out the many facets of adenine in coordination, crystal patterns, and catalysis.^[^ ^197^ ^]^ More than 1000 crystallographic purine‐related structures were added to the Cambridge Crystallographic Database (CCD) in the last 10 years. In these structures, the nitrogen atoms of the purine bicycles are involved in the formation of many metallic complexes (Ca, Co(II), Co(III), Cu(I), Cu(II), Mg, Mo, Ni, Zn, etc.).^[^ ^109^ ^]^ The unique versatility as metal ion ligand of adenine has been highlighted in a review article published in 2012 in comparison to hypoxanthine, xanthine, and guanine^[^ ^198^ ^]^ and, therefore, adenine appeared also to be very attractive as a substitute, under prebiotic conditions, of the imidazole ligand, especially in metallic binuclear complexes.

**Scheme 31:**
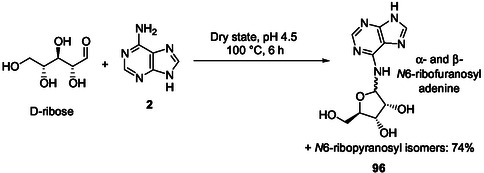
Reaction of adenine with D‐ribose at the dry state giving mainly N6‐ribosyl adenosine isomers.^[^ ^199^ ^]^

Here, we summarized works illustrating the potential catalytic properties of adenine **2** and N6‐substituted adenine derivatives under plausible prebiotic conditions. The low solubility of adenine in water limits its concentration in aqueous solution. 6‐Ribosyladenine isomers (α‐ and β‐N6‐ribofuranosyl derivatives **96** and the corresponding α‐and β‐N6‐ribopyranosyl isomers) are mostly formed when a dry mixture of adenine and D‐ribose is heated at 100 °C (**Scheme** **31**).^[^ ^199^ ^]^ Under these plausible prebiotic conditions, D‐ribose also reacts with guanosine 2‐amino group and, probably, with guanine to form the corresponding 2‐ribosylamino purine derivatives while hypoxanthine fails to react.^[^ ^199^ ^]^

**Scheme 32:**
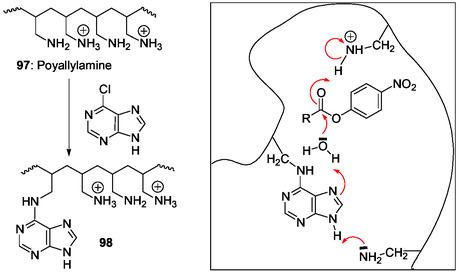
Preparation, from polyallylamine **97**, of polymers **98** incorporating the adenine bicycle attached by 6‐amino group and mechanism proposed for *p*‐nitrophenyl ester hydrolysis catalyzed by polymers **98** (R = methyl, propyl or *N*‐carboxybenzyl‐L‐alanyl).^[^ ^203^ ^]^

Thereby, N6‐ribosyladenosine isomers appeared to be good candidates to retain a part of the acid–base catalytic activity of the histidine imidazole ring. In the model reaction of 4‐nitrophenyl acetate hydrolysis (pH 7.7, room temperature), their catalytic efficiencies were found lower than that of histidine (relative efficiencies: 0.6 for N6‐isomers and 1.0 for histidine).^[^ ^200^ ^]^ To increase the observed catalytic effect of the adenine bicycle in ester hydrolysis, polyallylamine (**97**) polymers incorporating adenine grafted by its 6‐amino group **98** were synthesized (**Scheme** **32**).^[^ ^201^, ^202^, ^203^ ^]^ In these polymers, aliphatic protonated and deprotonated amine groups, present under slightly basic conditions, could participate to an acid–base ester catalysis. Hydrophobic dodecyl group was also grafted on the polymers to create a hydrophobic microenvironment favorable to binding of lipophilic substrate esters. These polymers exhibit pronounced catalytic activities in the model reaction of 4‐nitrophenyl acetate and 4‐nitrophenyl butanoate (PNB) hydrolysis. In mild basic conditions (pH 8.0), a 400‐fold acceleration of the PNB hydrolysis rate in comparison to adenine revealed a cooperative effect in proton transfer between the aliphatic amino groups and the adenine rings of the polymers (Scheme 32).^[^ ^203^ ^]^ The synthesized polymers are able to catalyze the hydrolysis of *N*‐carboxybenzyl‐L‐alanine 4‐nitrophenyl ester as well as 4‐nitrophenyl acetate and butanoate. The corresponding modeled kinetics displays a positive cooperativity.^[^ ^204^ ^]^ Such catalytic effects illustrate the potential role in catalysis under plausible prebiotic conditions of the adenine ring incorporated in RNA after ribosylation at N6.

**Scheme 33:**
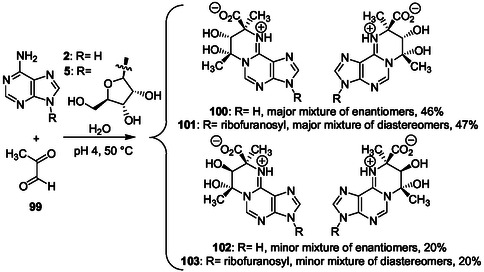
Structure of the condensation products of pyruvaldehyde (methylglyoxal) **99** on adenine **2** and adenosine **5**.^[^ ^206^ ^]^

**Scheme 34:**
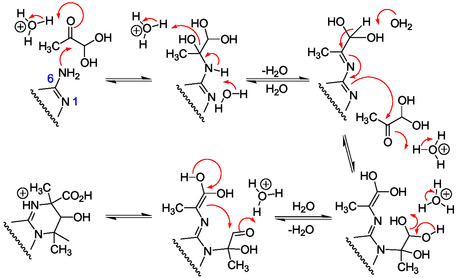
Proposed mechanism of condensation of two molecules of the pyruvaldehyde hydrate, present in water, on adenine and adenosine and α‐aminoazaheterocycles giving the adducts **100** and **101** in aqueous solution at pH 4 and 30 or 50 °C (unpublished mechanism).

In the search for new prebiotic reactions of adenine, under plausible prebiotic conditions, the reactions of adenine **2** and adenosine **5** were studied with pyruvaldehyde (methylglyoxal) **99**, the reduced form of pyruvate (Scheme 33). Adenine and adenosine were heated with 7 equivalents of pyruvaldehyde (methylglyoxal) in aqueous solution at pH 4 and 50 °C, for 18 h, under argon atmosphere or in the presence of dioxygen.^[^ ^205^, ^206^ ^]^ A remarkable stereoselective N1‐N6 cyclization resulted from reaction of adenine or adenosine with two pyruvaldehyde molecules and gave highly functionalized tricyclic adducts carrying a carboxylic acid function and two hydroxyl groups.^[^ ^206^ ^]^ From adenine **2**, two mixtures of diastereomers **100** and **102**, each corresponding to a racemic mixture of enantiomers, were isolated separately in 46% and 20% yields, respectively, and were characterized (**Scheme** **33**). The same reaction occurs also at pH 5 and 8 and at 30 °C. From adenosine **5**, the corresponding mixtures of diastereomers **101** and **103** were isolated in 47 and 20% yields. Due to the chirality of adenosine, two diasteromers, difficult to separate, were present in both isolated mixtures (not drawn in Scheme 33). This reaction was generalized to polyA and to numerous aromatic α‐aminoazaheterocycles such as 2‐aminopyridine, cytosine, and 1‐aminoisoquinoline.^[^ ^207^, ^208^ ^]^ A mechanism of the remarkable stereoselective reaction of adenine and adenosine with pyruvaldehyde is proposed in **Scheme** **34** (unpublished).

The catalytic efficiencies of both mixtures of enantiomers isolated from adenine have been measured in the 4‐nitrophenylacetate hydrolysis at pH 7.7 and 20 °C and were found to be close to the efficiency of histidine (relative efficiencies: 0.8, 0.9, 1.0, and 0.55 for **100**, **101**, histidine, and adenine, respectively).^[^ ^205^ ^]^ These results demonstrate the catalytic capability of N6‐alkylated and N1,N6‐dialkylated adenine derivatives in ester hydrolysis. The isolated adenine‐pyruvaldehyde adducts **100** and **101** are appeared also to be much more soluble in water than adenine. Under basic conditions, in 0.1 m aqueous NaOH, at room temperature, these adducts were completely and selectively converted to adenine, potentially through Dimroth rearrangement (unpublished results). Therefore, the observed easy formation of adenine N1,N6‐cyclic adducts from pyruvaldehyde can be seen, in a prebiotic context, as a mode of adenine solubilization and, interestingly, as a mode of reversible protection of the reactive adenine N1/N6‐amino functions allowing reactions at N9. The corresponding cyclization represents also a remarkable source of stereoselectivity. In the continuation of this approach, RNA aptamers able to complex free adenine were isolated using a systematic evolution of ligands by exponential enrichment (SELEX) procedure. A new purine binding motif was functionally and structurally characterized showing that the imidazole moiety is not trapped in the binding site, and would easily be available for catalysis.^[^ ^209^ ^]^ Interestingly, reaction of adenine N6 at a RNA abasic site would allow incorporation of N6‐ribosyladenine into RNA.

## 5 Conclusion

**Scheme 35:**
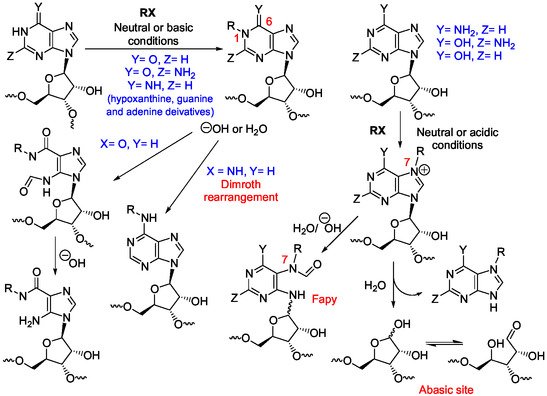
Main N‐alkylation reactions of purine ribonucleos(t)ide and corresponding opening of the imidazole or pyrimidine ring, and corresponding deglycosylation, reactions that can occur in primitive RNA under plausible prebiotic conditions.

Hypoxanthine, adenine, and guanine, present in RNAs and in free ribonucleos(t)ides, are made of four or five reactive nitrogen atoms that made them sensitive to many chemical modifications under plausible prebiotic conditions. N‐alkylation and N‐acylation of these nucleobases allow opening of the pyrimidine or imidazole ring under acidic, neutral, or basic aqueous conditions to give new imidazole derivatives and/or new pyrimidines such as formamidopyrimidines (Fapys) (**Scheme** **35**). Deglycosylation, abasic site formation, and RNA cleavage also result from N‐alkylation of purines in ribonucleos(t)ides and RNA, respectively, mainly through formation of unstable N7‐alkylpurinium products (Scheme 35).^[^ ^67^, ^210^ ^]^

*N* ^1^‐(β‐D‐Ribofuranosyl)‐5‐aminoimidazole‐4‐carboxamide AICAR **10** is a metabolite common to the purine ribonucleotide and histidine biosynthetic pathways. Interestingly, all atoms of AICAR produced by the histidine pathway have as origins ATP **11** except for the oxygen atom of the amide function originating from a water molecule. The corresponding biotransformation involves a first step of N1‐ribosylation of the adenine bicycle of ATP and, then, opening of the corresponding pyrimidine ring in PRAM **27** induced by addition of a water molecule at C6 and catalyzed by PRAMP cyclohydrolase that is a Mg^2+^–Zn^2+^ metalloprotein.^[^ ^72^, ^186^ ^]^ N1‐ or N7‐ alkylation of hypoxanthine, adenine, and guanine ribonucleos(t)ides occurs in aqueous solution under plausible prebiotic conditions. The produced N1‐alkyl inosine derivatives decompose, in aqueous solution under neutral or basic conditions, to give imidazole derivatives that are AICAR analogs.^[^ ^168^, ^169^ ^]^ Under basic conditions, N1‐alkyl adenosine derivatives undergo the Dimroth rearrangement to give the corresponding N6‐isomers. N6‐glycinyl adenine derivatives **58** and **61** and, probably, other N6‐α‐amino acyl adenine and adenosine derivatives, also decompose through Dimroth rearrangements to give AICAR analogs in neutral or basic aqueous solution.^[^ ^141^, ^142^, ^143^ ^]^ In strongly acidic aqueous solution, heating of 1‐methyladenosine led to 5‐aminoimidazole‐4‐*N*′‐methylcarbamidine, structurally related to AICA **40**, resulting from deglycosylation, addition of a water molecule and pyrimidine ring‐opening, and, then, loss of C2‐atom through hydrolysis of the intermediate Fapy derivative.^[^ ^66^ ^]^ Unfortunately, the behavior of N1‐ribosyl and N1‐alkyl adenosine derivatives in aqueous solution under mild neutral and slightly acidic or slightly basic conditions was not investigated to observe pyrimidine ring‐opening by cleavage of the N1‐C6 bond under plausible prebiotic conditions.

Clearly, the works summarized here demonstrate that adenine, hypoxanthine, and guanine, and the corresponding ribos(t)ides are sources of AICA and AICAR analogs and suggest that purine ribonucleotides can be converted under plausible prebiotic conditions to AICAR and/or related metabolites involved in the biosynthetic pathway of IMP such as CAIR **18**, SAICAR **19,** and FAICAR **20**, (Scheme 1).

DNA^[^ ^175^, ^211^ ^]^ and RNA^[^ ^63^, ^64^, ^65^, ^210^ ^]^ are permanently damaged by ultraviolet light, oxidation, chlorination, nitration, and alkylation and, DNA is repaired continuously. In cells, mRNA lifetime control limits the role of such modifications which were certainly key in the RNA world. Into the ribosome, ribosomal RNAs are protected by ribosomal proteins, at least in part, tRNAs that are hypermodified can be damaged and RNA also can be repaired.^[^ ^212^ ^]^ In DNA, many damages of pyrimidine and purine nucleic bases were identified, especially purine damages. Probably, reactions of the purine bicycle in primitive RNAs and ribot(s)ides with alkylating agents (electrophilic species) and acylating amino acids, for example, have generated numerous new heterocycles. The produced damaged purines, in nucleos(t)ides and/or in RNA, could have contributed to the emergence of different biosynthetic pathways such as the purine ribonucleotide and the histidine pathways through further chemical reactions. In the RNA world, repair enzymes can also emerge. IMP cyclohydrolases, catalyzing the final step of de novo purine biosynthesis to form IMP from FAICAR (5‐formylAICAR, Scheme 1)^[^ ^56^ ^]^ and GTP hydrolases allowing cleavage of the guanine imidazole ring^[^ ^176^, ^177^, ^180^, ^186^, ^213^, ^214^ ^]^ (Scheme 23), are perhaps vestiges of primeval RNA repair and/or RNA modification enzymes.

In primitive RNA and free nucleos(t)ides, conjugation of nucleobases to first amino acids and derivatives probably occurred by many ways under plausible prebiotic conditions (for example, condensation through wet–dry cycles). Some of the resulting modified pyrimidines and purines are probably molecular fossils present in contemporary tRNAs such as *N*6‐threonylcarbamoyladenosine (t6A).^[^ ^53^, ^154^, ^159^, ^160^ ^]^ The reported surprising transformation of *N*6‐glycinyladenine **58** and *N*6‐glycinyl‐9‐methyladenine **61** through Dimroth rearrangements to give *N*6‐carboxymethyl adenine derivatives **59** and **63**, respectively, well illustrate the rich molecular evolution offered by the purine chemistry (Scheme 9, 10, 15). Remarkably, this transformation, that should be general for α‐amino acids N6‐adenine and adenosine conjugates, results in production of ammonia/ammonium ion in neutral or slightly basic aqueous solution.^[^ ^141^, ^142^, ^143^ ^]^

Alkylation on the imidazole ring at N7, in adenosine, guanosine, and inosine, is also possible under plausible prebiotic conditions, in acidic, neutral, or basic aqueous solutions. It can result in the opening of the imidazole ring of the purine bicycle to give formamidopyrimidines (Fapys) and, then, the corresponding diaminopyrimidine ribonucleotide derivatives. The GTP Fapy metabolite **81** (Scheme 23) is involved in the biosynthetic pathways of biopterins, folate, riboflavin, and corresponding enzymatic cofactors. Formation of Fapys **83**, analogs of **81**, from guanosine and related ribonucleos(t)ides and/or in RNA, through reaction at C8 of guanine with hydroxyl radical under abiotic conditions (Scheme 24), was related to the emergence of corresponding cofactors.^[^ ^181^, ^182^, ^183^ ^]^ Such Fapy‐G derivatives should have been generated in primeval RNA like many other purine chemical lesions. The easy formation of N1,N6‐cyclic adducts of pyruvaldehyde to adenine and adenosine in aqueous solution under mild conditions, is another example of adenine and adenosine modification, giving adenine derivatives highly soluble in water and source of stereoselectivity (Scheme 33 and 34).^[^ ^206^, ^207^ ^]^ Purine nucleos(t)ide deglycosylation and abasic site formation in RNA^[^ ^67^, ^210^ ^]^ can also result from purine N7‐alkylation. However, such a deglycosylation occurs at a much slower rate for N7‐alkylated ribonucleos(t)ides and RNA in comparison to the corresponding modified deoxyribonucles(t)ide and DNA lesion. DNA abasic sites are unstable and undergo strand cleavage.^[^ ^211^ ^]^ Abasic RNA is significantly more stable than abasic DNA under different conditions.^[^ ^67^, ^210^ ^]^ The aldehyde function present at RNA abasic sites (Scheme 21 and 35) has thus time for reactions with other reagents such as amino acids and peptides avoiding RNA cleavage. Such RNA stabilizing effects, due to the presence of the 2’‐hydroxyl function,^[^ ^66^ ^]^ probably increased the number and nature of the chemical modifications present in primeval RNA and could be related to the emergence of first steps of metabolic‐like pathways especially in relationships with amino acid modifications such as their oligomerization. Furthermore, abasic sites also may be considered as sites for ribosylation allowing incorporation of noncanonical bases into RNA and for base exchanges.

The progressive release, from damaged RNA, of modified purines, nucleotides, and purine substituents such as amino acid and peptide derivatives, probably has happened through hydrolysis of modified adenines at 6‐amino group (and cytosines) and, also, through RNA hydrolytic cleavage. Indeed, also due to the presence of the 2’‐hydroxyl function, hydrolysis of RNA phosphodiester bonds occurs under basic conditions or catalyzed by metal ions and, results in the production of modified ribonucleotides and oligoribonucleotides and, in building block recovering for recombination. Such a hydrolytic cleavage can be favored by mispairing of the damaged bases and into nonhybridized base sequences due to reduced electronic density around phosphorus atoms. Recently, Gold et al. argued that noncanonical ribonucleotides, which would have been inevitable under prebiotic conditions (that may correspond to damaged nucleobases), might decrease the RNA length required to have useful catalytic function by allowing short RNAs to possess a more versatile collection of folded motifs.^[^ ^215^ ^]^

The investigation, under plausible prebiotic conditions, of reactions of amino acids adducts to canonical and noncanonical purine ribonucleos(t)ides (especially at N6 of adenosine) and, then, with RNA, should highlight some key chemical steps involved in the emergence of a primitive metabolism from a RNA world and through a RNA‐peptide world.^[^ ^51^, ^52^, ^53^, ^54^, ^55^, ^56^, ^57^, ^58^, ^59^, ^60^, ^61^, ^62^ ^]^ In this latter, probably, RNA and amino acids have been conjugated covalently by different chemical links to build and enrich in concert a primary metabolism. Progressively, benefiting from formation of new secondary and tertiary structures and new catalysts,^[^ ^215^, ^216^ ^]^ the macromolecular chemical library resulting from RNA chemical modification and peptide formation had to self‐organize.^[^ ^217^, ^218^, ^219^, ^220^, ^221^, ^222^ ^]^ Many reactions of purines with amino acids are possible under plausible prebiotic conditions, in RNA and from free ribonucleos(t)ides, and should be explored in the search for ways allowing the emergence of biosynthesis pathways.

## Conflict of Interest

The authors declare no conflict of interest.
